# Intrinsic edge dislocations promote high-temperature strength and ductility in additively manufactured refractory high-entropy alloys

**DOI:** 10.1038/s41467-026-71995-8

**Published:** 2026-04-24

**Authors:** Chunhuan Guo, Bo Jiao, Fengchun Jiang, Wei Chen, Wenyuan Wang, Cheng Zhang, Bozhao Zhang, Huabing Gao, Tao Dong, Wenyao Sun, Zubin Chen, Haixin Li, Zhenlin Yang, Shiteng Zhao, Jun Ding, Robert O. Ritchie

**Affiliations:** 1 College of Materials Science and Chemical Engineering, Harbin Engineering University, Harbin, China; 2 Shandong Key Laboratory of Additive Manufacturing Technology & Equipment, Yantai, China; 3 Harbin Engineering University Yantai Graduate School, Yantai, China; 4 Tianmushan Laboratory, Hangzhou, China; 5 Center for Alloy Innovation and Design, State Key Laboratory for Mechanical Behavior of Materials, Xi’an Jiaotong University, Xi’an, China; 6 Department of Materials Science and Engineering, University of California, Berkeley, Berkeley, CA USA

**Keywords:** Metals and alloys, Mechanical properties

## Abstract

Refractory high-entropy alloys (RHEAs) hold promise for applications in extreme environments. However, conventional as-cast RHEAs are constrained by the trade-off between strength and ductility, necessitating time- and energy-intensive post-processing. Here, we propose a streamlined strategy to fabricate RHEAs via laser directed energy deposition (LDED) using elemental powder blends, eliminating the need for post heat treatments. The additively manufactured (AMed) Nb_40_Ta_25_Ti_15_Hf_15_Zr_5_ alloy, characterized by a high density of intrinsic edge dislocations introduced during the thermal cycling of the process, demonstrates a remarkable tensile strength of ~497.3 MPa and a uniform elongation of ~6.8 % at 1000 °C, representing a ~ 37.8% and ~61.9% increase, respectively, over its as-cast counterparts. It is found that the intrinsic edge dislocations generated during AM process significantly enhances the alloy’s strain hardening capability at elevated temperatures. Simultaneously, the high density of edge dislocations effectively enhance material deformability through kink band formation and the stochastic nature of dislocation motion. This work presents a cost-effective pathway for the rapid fabrication of AMed RHEAs with an exceptional combination of high-temperature strength and ductility, paving the way for next-generation structural alloys in extreme environments.

## Abstract (web summary)

Researchers show that additive manufacturing can create high-density stable edge dislocations in a refractory high-entropy alloy, enabling unusually strong yet ductile performance at high temperatures and providing a new route to extreme-environment materials.

## Introduction

Single-phase body-centered cubic (BCC) refractory high-entropy alloys (RHEAs) have emerged as potential candidates for the next generation of high-temperature structural applications^1,2^. Substantial research has been dedicated to enhancing the processability and mechanical performance of RHEAs^3–5^. However, most of these advancements rely on conventional casting methods, which present several challenges. It necessitates multiple cold-rolling and complex heat treatments to achieve the pre-designed microstructure and excellent mechanical properties. Moreover, the vast compositional space of RHEAs makes it difficult to design alloys with precisely tailored properties using traditional casting techniques.

Additive manufacturing (AM) has garnered significant attention since it can overcome the limitations of conventional casting methods. The high dislocation density and refined grain structures in alloys impart superior mechanical properties^6–8^. In addition, AM has emerged as a high-throughput approach to fabricate and screen RHEAs by directly using elemental powder blends instead of costly pre-alloyed powders^9–12^. This capability provides an efficient platform for the development and investigation of high-performance RHEAs over a vast compositional space. To date, the in-situ multi-metal alloying AM has been used to develop various systems exhibiting exceptional strength, such as NbTiHf^13–16^, NbTaMo^17–20^, NbTaTi^21–23^ and so on. Among them, only AMed Ta-free alloys, such as NbTiHfZr^13^, Ti_42_Hf_21_Nb_21_V_16_^14,15^, and Ti_40_Hf_10_Nb_12_V_38_^16^, have exhibited appreciable room-temperature tensile ductility. However, it is difficult to fabricate full-size Ta-containing RHEAs due to the high melting point of Ta^17–22,24^, limiting mechanical property measurement to compression. Even in conventionally arc-melted Ta-containing RHEAs, this size limitation persists^25,26^. Therefore, the key challenge lies in successfully fabricating large-scale Ta-containing RHEAs while elucidating the fundamental mechanisms governing their strength-ductility balance.

In conventional single-phase BCC alloys, edge dislocations are generally more mobile than screw dislocations due to the thermally activated nucleation of screw dislocation kink pairs^27,28^. However, the unique atomic environment in BCC RHEAs leads to a fundamental alteration in dislocation mobility by creating a complex energy landscape for dislocation motion^29–31^. Consequently, compared to screw dislocations, the atomic environment-induced reduction in edge dislocation mobility persists across a wide temperature range, which significantly contributes to the strengthening behavior of RHEAs^32–35^. In addition, edge dislocations are also of benefit to strain accommodation and damage tolerance due to stress concentration reduction created by the formation of kink bands during deformation^36^. It is well known that the aforementioned theories of RHEAs have been predominantly developed based on fully recrystallized as-cast states with low dislocation densities. Therefore, these casting alloys exhibit limited strain hardening capability at elevated temperatures due to insufficient dislocation multiplication^36,37^. Conversely, in AMed RHEAs, a high density of intrinsic dislocations is introduced during the rapid solidification process, which can play an active role in strengthening and plasticity during deformation^38,39^. However, their contributions to mechanical performance, particularly at elevated temperatures, remain inadequately understood. The lack of experimental evidence on the temperature-dependent deformation mechanisms of AMed RHEAs represents a critical gap in current research.

Here, we propose an efficient and multifunctional laser-directed energy deposition (LDED) strategy to fabricate RHEAs while achieving near-homogeneous alloying of multiple refractory elements, even with melting point differences of up to 1400 °C (i.e., Ti and Ta as components in the current RHEAs). It has been confirmed that the intrinsic edge dislocations formed during the AM process exhibit excellent stability across a wide temperature range, significantly improving high-temperature strength and ductility while maintaining the toughening effect of kink bands. This investigation establishes a paradigm in AMed RHEA study, demonstrating that intrinsic dislocation structures can be harnessed for exceptional high-temperature performance without any post-processing.

## Results and discussion

### Additively manufactured Nb_40_Ta_25_Ti_15_Hf_15_Zr_5_ alloy

**Fig. 1:**
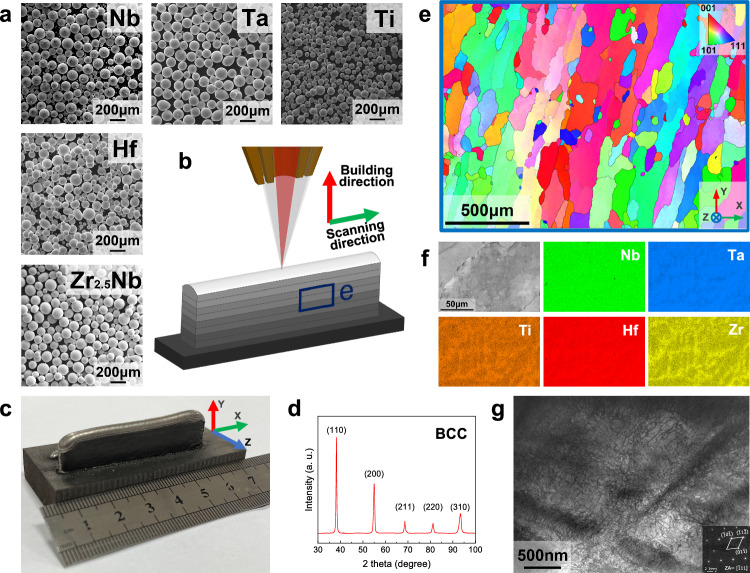
Preparation and initial characterization of Nb_40_Ta_25_Ti_15_Hf_15_Zr_5_ alloy fabricated by laser-directed energy deposition. **a** SEM images of Nb, Ta, Ti, Hf, and Zr_2.5_Nb spherical powders with a diameter of 53–150 μm. Due to the explosive nature of Zr powder, Zr_2.5_Nb powder was used as a safer alternative. **b** The schematic diagram of the LDED process, with a laser spot diameter of 1.3 mm, and a deposition layer thickness of 0.2 mm. **c** A thin-walled Nb40 specimen with dimensions of 50 × 11 × 3.5 mm^3^. **d** XRD indicates the single BCC phase structure. **e** EBSD IPF map of the XY cross-section of AM-Nb40, with the scanning area shown in (**b**). **f** The band contrast (BC) micrograph and energy-dispersive X-ray spectroscopy (EDS) images of the local region in AM-Nb40. **g** Bright field (BF) TEM image of AM-Nb40 sample showing a high density of intrinsic dislocations; the inset is the selected area electron diffraction (SAED) pattern.

A non-equiatomic Nb_40_Ta_25_Ti_15_Hf_15_Zr_5_ (at. %, abbreviated as Nb40) RHEA was chosen as the model alloy for this study (Supplementary Note 1). The raw materials include spherical powders of Nb, Ta, Ti, Hf, and Zr_2.5_Nb with a purity exceeding 99.95% (Fig. 1a). The samples were fabricated on pure Ti substrates using the LDED system (Fig. 1b). This study develops a gradient in-situ alloying LDED strategy to address critical challenges in AM of refractory powders. This strategy offers three main advantages: (i) implementation of backing and transition layers to prevent substrate burn-through and mitigate Ti segregation through gradually increased heat input; (ii) optimization of a remelting process with appropriate power density and scanning speed to ensure complete melting of refractory powders and bulk homogenization; (iii) formulation of a systematic protocol for parameter optimization in layer-by-layer deposition. This strategy (detailed in “Methods” and Supplementary Note 2) successfully overcomes the inherent difficulties of elemental powder melting and substrate compatibility, as confirmed by defect-free fabrication demonstrated in an optical microscopy (OM) image (Supplementary Fig. 1a).

Figure 1c displays a 50 × 11 × 3.5 mm^3^ thin-walled specimen without any surface cracks. The OM images of AMed Nb40 (AM-Nb40) in Supplementary Fig. 1b–d reveal typical dendritic and interdendritic microstructures, which result from the differences in melting points of the constituent elements. The energy-dispersive X-ray spectroscopy (EDS) mapping in Fig. 1f confirmed that the dendrite regions are rich in Ta, and the interdendritic regions are rich in Ti and Zr. X-ray diffraction (XRD) revealed that the AM-Nb40 alloy is composed of a single BCC phase (Fig. 1d). Figure 1e shows a coarse columnar grain morphology along the building direction (BD) with some partially recrystallized grains. As shown in Supplementary Table 1, the actual compositions of AM-Nb40 closely match the nominal compositions. The microstructure and chemical homogeneity are detailed in Supplementary Note 3. In addition, transmission electron microscopy (TEM) analysis indicated that the rapid solidification of LDED generates a high density (~6.26 × 10^13^ m^−2^) of pre-existing dislocations in AM-Nb40 (Fig. 1g). To directly demonstrate the effect of intrinsic dislocations on mechanical performance, the fully recrystallized as-cast Nb40 samples (FR-Nb40) were fabricated with low initial dislocation density (detailed in Methods and Supplementary Fig. 2a, b).

**Fig. 2:**
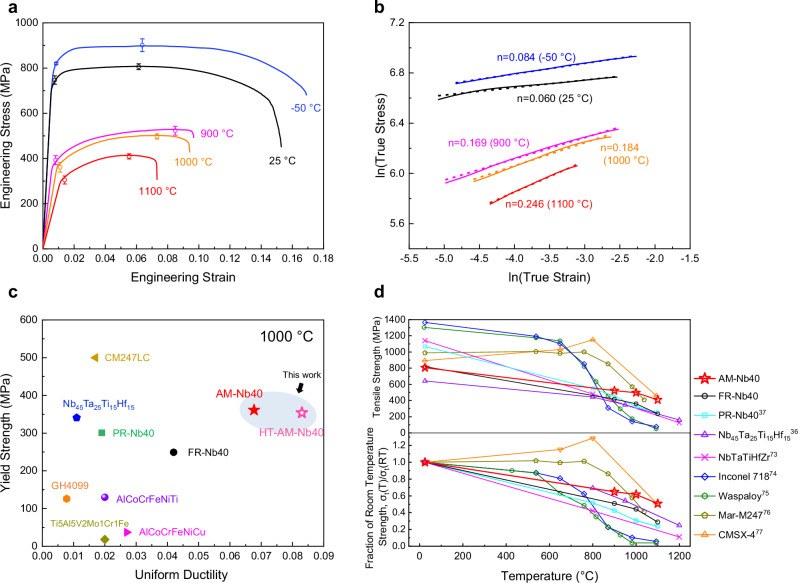
Tensile properties of AM-Nb_40_Ta_25_Ti_15_Hf_15_Zr_5_ RHEA. **a** Tensile engineering stress-strain curves measured from −50 to 1100 °C. The curves represent the typical experimental results at each temperature. The yield strength (σ_y_) and tensile strength (σ_t_) are marked on the curves, whereas the data points are means ± standard deviation of three tests. **b** Strain hardening exponents obtained at each temperature. Compared to low temperature properties, the alloy has higher strain hardening exponents at elevated temperatures. **c** Yield strength *vs*. uniform elongation plots of AM-Nb40, FR-Nb40, other RHEAs with similar compositions, and commercial materials at 1000 °C^36,37,68–72^. (The testing temperature for the Nb_45_Ta_25_Ti_15_Hf_15_ and Ti5Al5V2Mo1Cr1Fe alloys are 950 °C.) The AM-Nb40 has an exceptional synergy of yield strength and uniform elongation at elevated temperatures. **d** Tensile strength *vs*. temperatures plots of AM-Nb40, commercial Ni-based superalloys and RHEAs^36,37,73–77^. AM-Nb40 has moderate strength and elongation at room temperature, but excellent strength and strength retention at high temperatures. The high-temperature performance is superior to most commercial high-temperature materials. All detailed data are presented in Supplementary Table 2.

We evaluated the mechanical properties of the AM-Nb40 and FR-Nb40 by performing uniaxial tensile tests at temperatures ranging from −50 to 1100 °C. Engineering stress-strain curves of the AM-Nb40 (Fig. 2a) show that at room temperature, the yield strength (σ_y_) is 747.0 ± 18.6 MPa with a fracture elongation of 14.8 ± 0.7%, both strength and elongation are lower than FR-Nb40 (Supplementary Fig. 2c). At −50 °C, both alloys exhibit identical σ_y_ of ~820 MPa, while the elongation of AM-Nb40 is significantly higher than that of FR-Nb40. With increasing temperature, σ_y_ values of AM-Nb40 are 392.5 ± 19.8 MPa (900 °C), 361.1 ± 21.3 MPa (1000 °C), and 304.9 ± 17.7 MPa (1100 °C), all significantly higher than those of the FR-Nb40. Moreover, at all these temperatures, tensile plasticity of AM-Nb40 is quite evident. As shown in Supplementary Fig. 3, the fracture surfaces of AM-Nb40 at all investigated temperatures primarily consist of ductile dimples (microvoid coalescence). Although a few river patterns and cleavage steps can be observed in the post-deformed samples at high temperatures, the widespread presence of dimples indicates that plastic deformation is still the main deformation mode, which is not common in AMed RHEAs at these temperatures.

Beyond the above performances, the AM-Nb40 exhibits significant strain hardening capability at high temperatures. Figure 2b demonstrates the strain hardening exponent (*n*) increases from 0.060 (25 °C) to 0.246 (1100 °C) with increasing temperature, which is like that of FR-Nb40 (Supplementary Fig. 2d). However, AM-Nb40 exhibits a better combination of yield strength and uniform elongation at elevated temperatures, outperforming RHEAs with similar compositions and conventional high-temperature materials (Fig. 2c). Meanwhile, the tensile strength (σ_t_) is clearly superior due to the continuous high work hardening. The σ_t_ of AM-Nb40 is compared with that of other RHEAs and that of the currently used commercial high-temperature materials in Fig. 2d for ambient to elevated temperatures. At temperatures exceeding 1000 °C, AM-Nb40 exhibits relatively higher strength compared to the RHEAs enriched with IV and V group elements and approaches the strength of Ni-based superalloys containing W or Re that were designed for blade applications. Notably, the σ_t_ of AM-Nb40 at 1000 °C exceeds that of the FR-Nb40 by approximately 37.8% (~136 MPa), demonstrating a remarkable achievement at such elevated temperatures. The AMed RHEAs in specific systems demonstrate exceptional strain hardening capacity at elevated temperatures. Compared to the as-cast counterparts, these alloys achieve simultaneous high strength and superior uniform elongation.

### Critical role of intrinsic edge dislocations

**Fig. 3:**
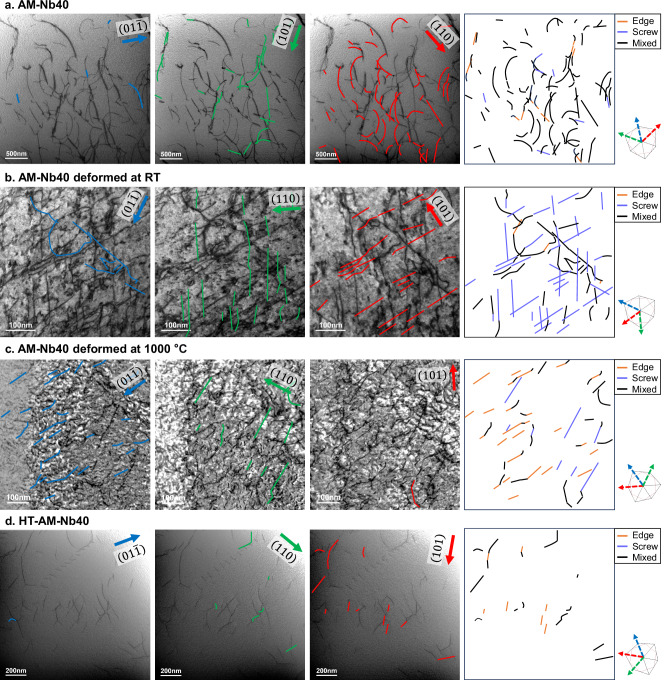
Dislocation types analysis via TEM. **a** Bright field (BF) images of AM-Nb40 sample. **b**, **c** BF images of AM-Nb40 RHEA after tensile deformation at room temperature and 1000 °C. **d** BF images of AM-Nb40 RHEA after annealing at 1000 °C for 1 h (HT-AM-Nb40). The spatial directions of the Burgers vectors are marked on the right side of the images, respectively. Schematics of dislocations colored by type: orange for edge dislocations, purple for screw dislocations, and black for mixed dislocations. Dislocations showing extinction under different g vectors are directly labeled in the corresponding BF micrographs using colors of their Burgers vectors. Detailed analysis methods and procedures are shown in “Methods” and Supplementary Fig. 4, respectively.

The dominant strengthening mechanisms in single-phase BCC RHEAs are closely related to the variations in dislocation mobilities^40^. Here, we examined the activated dislocation types of AM-Nb40 before and after tensile deformation using transmission electron microscopy (TEM). The zone axis is [$\bar{1}11$] or [$1\bar{1}\bar{1}$], and the $\vec{{{\bf{g}}}}$ vectors were determined by $\vec{{{\bf{g}}}}\,{{\boldsymbol{\cdot }}}\,\vec{{{\bf{b}}}}$ analysis to identify screw and edge dislocations. Figure 3 shows the extinction properties and dislocation types analysis of bright field (BF) images under different states (see Supplementary Fig. 4 for details). As illustrated in Fig. 3a, the dislocations are disorderly distributed in AM-Nb40. In addition to mixed dislocations, a minor fraction of edge and screw dislocations are identifiable. After tensile testing at room temperature, the dislocations primarily consist of long, straight screw dislocations (Fig. 3b), with the emergence of dislocation dipoles and loops, indicating the occurrence of cross slip^41^. This experimental observation suggests that edge dislocations exhibit greater mobility than screw dislocations at room temperature, aligning with the well-established kink mechanism of screw dislocations in BCC alloys. However, relative to FR-Nb40, the high density of intrinsic dislocations did not enhance the σ_y_ of AM-Nb40. Instead, the abundant cross-kinks accelerated the development of stress concentration, resulting in reduced elongation (Supplementary Note 4).

After high-temperature deformation, there is a clear reversal in the dominance of screw and edge dislocations (Fig. 3c). Unlike Fig. 3a, b, the proportion of edge dislocations in the alloy increased substantially. The mobility of certain edge dislocations was significantly suppressed at elevated temperatures compared to their behavior at room temperature^42^. This observation is consistent with previous findings on the temperature-dependent mobility of screw and edge dislocations in as-cast recrystallized alloys^29,32,33^. As the temperature increases, thermal vacancies overcome the hindrance of screw dislocations created by jogs/dipoles pinning^43^. Meanwhile, edge dislocations may encounter significant energy barriers during sliding. In contrast to fully recrystallized alloys, where dislocations primarily originate from source activation^44^, edge dislocations in AM-Nb40 may also derive from intrinsic dislocations generated during the AM process, which can directly participate in slip and multiplication processes. Therefore, the cooperative movement of intrinsic dislocations in AM-Nb40 plays a critical role in affecting their strengthening mechanisms and mechanical properties.

The key to maintaining high strain hardening performance during deformation lies in increasing migration resistance while enhancing dislocation multiplication^5,45,46^. Consequently, we have identified two critical factors to understand the high-temperature strain-hardening mechanism of intrinsic dislocations in this alloy. The first factor is the high-temperature stability of these dislocations. For this purpose, the AM-Nb40 alloy was heat-treated at 1000 °C for 1 h and subsequently water quenched (HT-AM-Nb40). As shown in Fig. 3d, the dislocation density in the RHEA markedly decreases at elevated temperatures, with all screw dislocations becoming efficiently exhausted due to dislocation annihilation. It is found that some edge and mixed dislocations remain in the matrix, which is rarely observed in annealed as-cast RHEAs. Previous studies^47–49^ have also shown such sluggish migration velocities and enhanced thermal stability afforded by intrinsic dislocations in AMed alloys with cellular structures. Despite the absence of a pinning effect from dislocation networks, intrinsic non-screw dislocations still have high-temperature stability.

The second factor is the retention rate of the dislocation density, which determines the ability of dislocations to propagate at elevated temperatures. The modified Williamson–Hall analysis method was employed to calculate dislocation density^50^ (Supplementary Fig. 5 and Note 5). As shown in Supplementary Table 3, the dislocation density in the AM-Nb40 was determined to be ~6.26 × 10^13^ m^−2^, a value comparable to that reported for AMed BCC alloy^51,52^. After 1000 °C tensile deformation and 1000 °C heat treatment, the values decreased to approximately ~2.53  × 10^13^ m^−2^ and ~9.14 × 10^12^ m^−2^, respectively, yet remained higher than those in FR-Nb40 (~8.93 × 10^11^ m^−2^).

In addition, HT-AM-Nb40 exhibited a yield strength of ~354.0 MPa and an ultimate tensile strength of ~506.3 MPa at 1000 °C, respectively, with a fracture elongation exceeding 11.5% (Supplementary Fig. 6). Notably, the strength of AM-Nb40 shows no significant reduction after the dislocation annihilation induced by the 1 h 1000 °C heat treatment, while ductility is further improved relative to the as-built AM state. The yield strength and uniform elongation of HT-AM-Nb40 remain notably higher than those of FR-Nb40 and many conventional high-temperature materials in Fig. 2c. This behavior is mechanistically significant because many conventional high-temperature alloys, despite their elevated fracture ductility through thermal softening, often suffer from insufficient strain hardening capacity at high temperatures, which results in premature necking. By contrast, in AM-Nb40, the intrinsic edge dislocations retained after high-temperature exposure provide substantial local slip resistance and thus sustain strength at 1000 °C. Sustained uniform plastic deformation requires continuous dislocation multiplication to stabilize plastic flow and prevent stress and strain concentrations. The AM-Nb40 maintains this substantial edge dislocation density and demonstrates continuous dislocation multiplication even at elevated temperatures. The mutual interactions between these intrinsic edge dislocations and deformation-induced dislocations from activated sources significantly enhance the work hardening behavior, thereby improving uniform elongation while maintaining high strength.

### Kink band formation

**Fig. 4:**
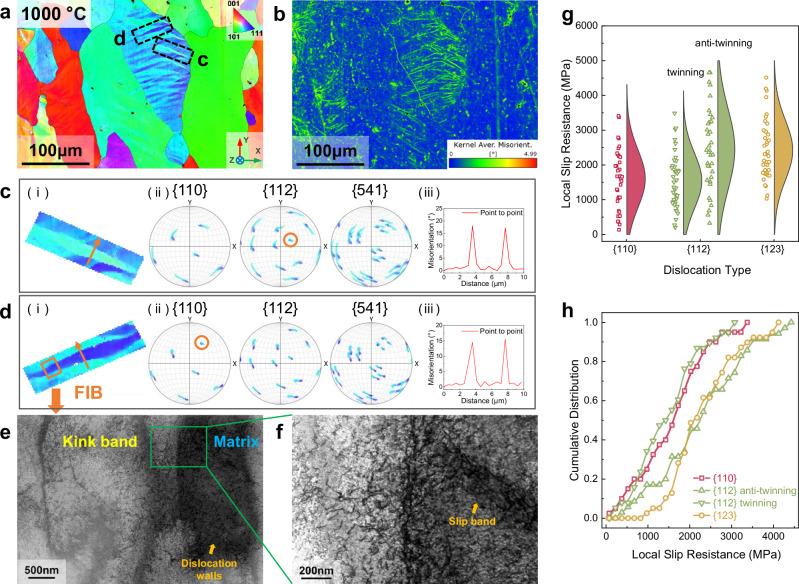
Kink band and local slip resistance (LSR) analysis. **a** The EBSD IPF + GB map of the tested sample deformed at 1000 °C**. b** Kernel average misorientation (KAM) map corresponding to (**a**). **c**, **d** (i) Two typical kink bands taken from the rectangular subsets in (**a**). (ii) Analysis of active slip systems. (iii) Misorientations along two arrow directions. **e** The BF TEM image of the {112} kink band segment in (**d**). **f** The locally magnified view of the rectangular area in (**e**). **g** Violin plots showing the distribution of LSR values for dislocations gliding on {110}, {112} (twinning and anti-twinning directions), and {123} planes, derived from molecular dynamics simulations. Each data point represents the LSR for a specific atomic configuration. **h** Cumulative distribution curves of LSR for the respective slip planes. The broad spread in LSR values highlights the stochastic nature of dislocation glide in the chemically complex matrix.

Furthermore, we conducted a comprehensive post-deformation characterization of AM-Nb40 samples using electron backscatter diffraction (EBSD) analysis to investigate the temperature-dependent microstructural evolution. Inverse pole figure (IPF) images are shown in Supplementary Fig. 7. At −50 to 1000 °C, the alloy maintains a typical columnar crystal structure (Supplementary Fig. 7a–d). At 1100 °C, the original columnar crystals are replaced by a dynamic recrystallization structure (Supplementary Fig. 7e). In contrast to the necklace-like recrystallized structure surrounding the equiaxed grains in annealed RHEAs during high-temperature deformation^53^, the high-density dislocations in AMed alloys provide the driving force for a fully recrystallized structure. At all testing temperatures, {332} < 113> twins are not observed, which is different from the as-cast Nb40^37^. Kink bands formed by concentration of edge dislocation arrays can be identified (Supplementary Fig. 7f–i), a phenomenon also extensively observed in deformation structures of other as-cast or AMed RHEAs^36,54,55^. This mechanism persists even at temperatures as high as 1000 °C (Fig. 4a), with nucleation exclusively in the regions of most severe necking and stress concentration. Local Kernel average misorientation analysis shows that most of the geometrically necessary dislocations (GNDs) are confined to the boundaries of the kink bands (Fig. 4b). Through subset pole figure analysis based on edge dislocation slip-induced lattice rotation theory, we identified the predominant slip modes. As shown in Fig. 4c, d, two kink bands within the same parent grain are activated by the {110} and {112} slip systems, respectively.

To directly observe their microstructure, we extracted a segment of the {112} kink band from the sample tensile-tested at 1000 °C using focused ion beam (FIB). As shown in Fig. 4e, the width of this kink band exceeds 2 μm. The high-density dislocation wall has formed at the kink band boundary, which is a direct product of dislocation slip and multiplication. Simultaneously, the slip band extends to the kink band boundary and terminates there (Fig. 4f). Slip bands are macroscopic traces of collective dislocation glide, and their termination at the kink band boundary indicates that dislocation motion is hindered and accumulates at this location^56^. This microstructural morphology directly demonstrates that, even at the high temperature of 1000 °C, active dislocation slip and multiplication occur at the early-stage boundaries of kink band nucleation, providing the dislocation foundation for boundary establishment. The dislocation walls constitute the embryonic form of the kink band boundary, while continuous dislocation activity (multiplication and accumulation) drives the gradual evolution of this boundary into a mature kink band interface with significant misorientation^56–58^. The kink bands continuously rotate the lattice to accommodate the exceptionally high strain near crack tips, effectively distributing damage. The results given in Supplementary Figs. 8–11 show that the variation in length, width, and cross nucleation of kink bands fundamentally stems from the interaction between tilt boundaries of {110}, {112} slip systems, imparting sufficient plasticity to the alloy. The lattice twist caused by the accommodation of kink bands under the exceptionally high strains leads to the simultaneous or alternating participation of previously inactive slip systems in deformation. This observation underscores the dynamic interplay between multiple slip systems in these alloys, highlighting the adaptability and coordination of the kink bands during deformation.

As demonstrated by the above analyses, the high-temperature plastic deformation of AM-Nb40 is governed by the synergistic interaction between dislocation slip and the formation of kink bands. The intrinsic edge dislocations promote strain hardening and uniform plastic deformation through their high slip resistance and multiplication mechanisms. The kink bands optimize the dislocation density and distribution, effectively accommodating the strain gradient to ensure sufficient strain hardening and compatibility for stabilizing uniform plastic flow, thereby preventing stress localization. The synergistic mechanism between strain hardening and stress relaxation at elevated temperatures confers AM-Nb40 with superior tensile strength and uniform elongation compared to the as-cast RHEAs (Fig. 2c). However, kink bands, which form typically through the exhaustion of slip systems, exhibit distinct activity differences from edge dislocations that persist across a wide temperature range. Consequently, it is crucial to understand the coexistence mechanisms of these dislocations at elevated temperatures.

### Stochastic nature of dislocations slip

To investigate the phenomenon of energy anisotropy in dislocation motion, the local slip resistance (LSR) of edge dislocations on the {110}, {112}, and {123} planes in Nb40 with random atomic ordering was evaluated using molecular dynamics (MD) simulations, as described in the Methods section. Short dislocation segments were modeled to glide by a single lattice periodicity within the alloy’s chemically heterogeneous environment. These simulations enabled the quantification of energy barriers encountered by dislocations on various slip planes. The results, presented in Fig. 4g, reveal a broad distribution of local slip resistance values for each slip plane. This distribution reflects the stochastic nature of dislocation motion in Nb40, where local atomic arrangements play a critical role in modulating glide resistance^30^. The chemically complex matrix creates a heterogeneous energy landscape^59^, resulting in variations in dislocation activity across different slip planes. Notably, low-activity edge dislocations contribute to strengthening by resisting glide, while high-activity dislocations enhance ductility by facilitating toughening. These competing effects are distributed among the {110}, {112}, and {123} planes, highlighting the intricate interplay between chemical heterogeneity and dislocation mechanics. Among the slip planes, {110} and {112} (twinning direction) consistently exhibit the lowest local slip resistance values, indicating a strong preference for dislocation glide along these planes under the specified conditions. This preference is attributed to the relatively lower energy barriers for dislocation motion, which strictly depend upon the favorable local atomic configurations along these planes. Figure 4h further illustrates the cumulative distribution of local slip resistance values, which quantifies the proportion of dislocations capable of overcoming specific resistance thresholds. The leftward shift of the cumulative distribution curves for {110} and {112} (twinning direction) planes reinforces their statistically lower glide resistance compared to those for the {112} (anti-twinning direction) and {123} planes.

These simulation results highlight the unique ability of Nb40’s chemical heterogeneity to modulate edge dislocation glide resistance, even on planes less favorable in simpler alloys. This behavior ensures that dislocations encounter a range of energy barriers and promotes a more uniform distribution of plastic deformation. Such properties are particularly beneficial in high-temperature environments, where localized stress concentrations often lead to premature failure. By enabling access to multiple glide planes, Nb40 enhances its damage tolerance and reduces the risk of catastrophic failure—a common limitation in conventional alloys. Furthermore, the alloy’s ability to balance low-resistance pathways for ductility with high-resistance barriers for strengthening enables it to achieve an optimal combination of mechanical properties. These findings provide valuable insights into the design of next-generation high-temperature structural materials by leveraging chemical complexity. This accelerates the path from alloy concept to application, particularly for components with complex geometries or operating under extreme conditions.

In summary, our work provides a more cost-effective and time/energy-efficient pathway for producing high-performance, geometrically complex RHEA components compared to traditional casting methods. Without requiring additional post-processing, the AM-Nb_40_Ta_25_Ti_15_Hf_15_Zr_5_ RHEA demonstrates exceptional mechanical properties at both room and high temperatures. The AM-generated intrinsic edge dislocations enhance the high-temperature strain hardening behavior significantly. Furthermore, multi-slip edge dislocations facilitate the formation of kink bands, thereby dispersing localized stress concentrations. The interplay between local atomic configurations and dislocation behavior in the alloy creates a complex yet advantageous landscape of dislocation pathways. This synergy ensures an optimal balance between strength and ductility, which makes AMed RHEAs a promising candidate for applications at elevated temperatures. Although further work is warranted to maximize the long-term stability of the intrinsic dislocation structures under prolonged thermal exposure, the present results establish a framework for the rapid development and screening of advanced RHEAs tailored for high-temperature applications through AM technology.

## Methods

### Powder blends

Nb, Ta, Ti, Hf, and Zr_2.5_Nb spherical powders were used as the raw materials to fabricate AMed Nb_40_Ta_25_Ti_15_Hf_15_Zr_5_ RHEA, which were produced using the Plasma Rotating Electrode Process (PREP). All powders have a purity of 99.95% (wt.%), and a size range from 53 to 150 µm, which is popular for LDED systems. The feedstock materials were purchased from Xi’an Hanhai New Materials Technology Co., Ltd. Zr powders present certain risks and have been associated with industrial accidents in the past^60,61^. We chose the stable Zr_2.5_Nb powders as a substitute. The particle size distribution, apparent density, tap density, and flowability of the powders before and after mixing are summarized in Supplementary Table 4. Each powder was weighed according to the mixed composition in Supplementary Table 1.

### LDED processing

Most studies on refractory high-entropy alloys (RHEAs) rely on traditional casting methods, which present several challenges. The significant differences in melting points and densities of refractory elements often result in severe elemental segregation, requiring multiple melting cycles and complex heat treatments for achieving compositional homogeneity. Moreover, the vast compositional space of RHEAs makes it difficult to design alloys with precisely tailored properties using traditional casting techniques.

The LDED technology offers significant advantages in the rapid design and screening of RHEAs, since it is convenient to implement high-throughput preparation using pre-mixed elemental powders or multiple independently controlled powders feeding^13,23,62,63^. However, there are still challenges in manufacturing RHEA through LDED process, as Ta powders have high melting points (Supplementary Table 1)^39^. It is easy to retain unmelted powder during the laser in-situ alloying process of mixed element powders^62,64^. In addition, there is a significant difference in thermal properties between the RHEA and the pure Ti substrate. High power can cause severe Ti segregation and defects such as melt through and holes on the substrate, which seriously affect the forming quality. Here, we propose an efficient and versatile LDED strategy for manufacturing RHEAs, enabling near-homogeneous alloying of multiple refractory elements with melting point differences as large as 1400 °C (e.g., Ti and Ta in the current RHEAs).

During the AM process, backing layer, transition layer, and remelting processes are implemented. The raw powders with a high melting point necessitate exceptionally high heat input to achieve complete melting. However, excessively high power can cause burn-through of the substrate and lead to excessive segregation of Ti from the substrate into the RHEA. For this purpose, six layers of mixed powders are coated on the substrate through low-power parameters in the initial stage of the AM process. These rough, non-homogeneous, and partially-melted layers are called backing layers, which serve to prevent burn-through. Subsequently, starting from the seventh layer onwards, the power gradually increases, and these layers are designated as transition layers. The transition layers mitigate the segregation of Ti elements through a gradual increase of thermal input.

Commencing from the transition layer of the 7th layer, the remelting process with higher power density is introduced. The remelting strategy offers more opportunities for the melting of high-melting-point refractory alloy elements, while also eliminating defects and reducing elemental segregation^9^. This strategy has been repeatedly implemented in research on in-situ multi-metal alloying additive manufacturing^11,14,51,64,65^. As illustrated by the optical microscopy images of the sample in Supplementary Fig. 1, the AM-Nb40 was fabricated without noticeable defects. This approach not only facilitates high-quality RHEA production but also provides a versatile framework for rapid development of refractory alloy systems via LDED.

The LDED system is equipped with an RFL-C12000X fiber laser, an ABB IRB2600 industrial robot, a powder feeder, and a cladding head. The laser was manufactured by Wuhan Raycus Laser Technologies Co., Ltd., with a laser wavelength of 1080 nm and a maximum output power of 12,000 W. The LDED process was carried out in an argon-protected atmosphere. The substrate is composed of pure Ti with dimensions of 100 × 200 × 10 mm^3^. The spot diameter is 1.3 mm; the cladding head is 20 mm away from the substrate. The thickness of each layer of deposition is 0.2 mm, and the cladding head is raised 0.2 mm each time. The optimized deposition parameters are detailed in Supplementary Figs. 12–14, Tables 5 and 6, and Note 2.

### Fabrication of reference samples

The cast RHEA ingots of identical composition were produced by arc-melting mixtures of high-purity raw materials (Nb, Ta, Ti, Hf, and Zr, all > 99.9 wt.%) under argon atmosphere. The raw materials were purchased from Laiyan Technology Hebei Co., Ltd. Prior to melting, residual oxygen was scavenged using a high-purity Ti getter. To promote homogeneity, each ingot was inverted and remelted 5–8 times. The as-cast samples (8–9 mm thick) were cold-rolled to 85% thickness reduction, followed by annealing at 1300 °C for 1 h in Ar-filled quartz tubes and subsequent water quenching to obtain a fully recrystallized microstructure, referred to FR-Nb40. The partially recrystallized as-cast Nb40 featuring a heterogeneous lamellar structure in a previous study^37^ has also been used for comparative analysis, referred to PR-Nb40.

In addition, the AM-Nb40 alloy was post heat-treated at 1000 °C for 1 h, followed by water quenching, referred to HT-AM-Nb40. It was characterized after tensile testing at high temperature to verify the thermal stability of the intrinsic dislocation structure in AM-Nb40.

### Composition analysis

Energy-dispersive X-ray spectroscopy (EDS) point scanning and microhardness tests were conducted to analyze the elemental compositions along the sample height (Supplementary Fig. 15), with detailed procedures provided in Supplementary Note 3. The results indicate that the composition stabilizes in regions above 4 mm in height. The nominal elemental composition of the AM-Nb40 and FR-Nb40 alloy, along with its oxygen and nitrogen contents measured using an oxygen/nitrogen detector, is summarized in Supplementary Table 1.

### Microstructural characterization

Optical microscopy (OM), X-ray diffraction (XRD), scanning electron microscopy (SEM), electron backscatter diffraction (EBSD), and transmission electron microscopy (TEM) were used to characterize the microstructure of AM-Nb_40_Ta_25_Ti_15_Hf_15_Zr_5_ RHEA. The rough specimens were cut from thin-walled specimens using a wire electrical discharge machine (EDM). These specimens were mounted in epoxy resin and mechanically mirror-polished for microstructural characterization. The samples were observed using a Zeiss Axioscope 5 OM. The surfaces of the samples were ground with 5000-grit SiC paper and polished with a diamond suspension to achieve a mirror finish. The samples were chemically etched in an etching solution of 5% hydrochloric acid, 15% nitric acid, and 80% distilled water. Additional characterizations were performed in the XZ section. Phase detection was carried out on the XRD equipment with Cu-Kα radiation, scanning angles ranging from 30° to 100° and a scanning speed of 1°/min. The elemental distribution and fractography of RHEA were determined by a SEM (Thermofisher APREO 2 C) equipped with an EDS system (Ultim Max).

A SEM (FEI Verios 5 UC) equipped with an EBSD detector (Symmetry) was used to investigate the microstructure in the RHEA samples. Grinding and ion-beam polishing were carried out at mid-thickness to enable the examination of crystallographic alterations induced by deformation through EBSD analysis. Step sizes of 3 µm and 0.5 μm were used for EBSD mapping. The average acquisition rate exceeded 300 Hz, and the indexing rate was above 90%. The mean angular deviation (MAD) was below 1°, indicating reliable indexing. Oxford AZtecCrystal software (2.1 version) was used to analyze the grain morphology, textures, and Kernel average misorientation of the RHEA before and after deformation. To analyze the kink band structure formed during high-temperature deformation, EBSD mapping was performed on the tensile fracture region (tested at 1000°C) to identify and delineate the kink band (Fig. 4c, d). A TEM lamella was then extracted from the targeted area using a focused ion beam (FIB) system (ZEISS Crossbeam 350). Dislocation analysis was performed before and after deformation using TEM (FEI Talos F200X). For the other TEM investigations, the samples were mechanically ground to a thickness of ~50 μm and then punched into a disk with a 3 mm diameter.

### Mechanical property measurements

The RHEA samples were machined into dog-bone shape for tensile tests, the dimensions are shown in Supplementary Fig. 16. All plate shaped specimens were ground to a mirror finish with 3000-grit SiC sandpaper. The room temperature tensile testing was performed using a universal testing frame (INSTRON 68MT-50) equipped with a video extensometer. The high-temperature tensile tests were conducted using an UZDL-2-2500 (Sinotest Equipment Co., Ltd.) system, which can provide testing temperatures ranging from 600 to 1700 °C. The deformation chamber was evacuated to 5 × 10^−3^ Pa with a vacuum pump connected to the chamber before heating up the specimen. Then, Ar gas was filled into the deformation chamber to prevent high-temperature oxidation of the RHEAs. The temperature increased using a resistance furnace. Water-cooled Molybdenum alloy fixtures were placed at both ends of the sample, and an extensometer was employed to measure the strain. The specimens were heated from room temperature to a given deformation temperature at a heating rate of 10 °C/s and held isothermally for 10 min. A B-type thermocouple spot-welded to the center of the sample and high-precision non-contact photoelectric colorimeter (infrared induction) were used to monitor and control temperature during testing. After the sample fractured, it cooled down to below 200 °C in the furnace. The −50 °C tensile test was conducted using an environmental chamber (Jilin Sandu Testing Equipment Co., Ltd.), which can provide a testing environment from −190 to 350 °C. The cooling copper tube of the environmental chamber was circulated with liquid nitrogen for cooling, and then kept cold for 10 min after cooling down. A PT100 sensor was used to measure temperature, with an error of ± 2 °C. Strain measurement was completed by a video extensometer. The strain rate of all tensile tests in the current study was set to 1 × 10^−3^ s^−1^. At least three tests were performed for the same temperature to ensure reproducibility of the results. The yield strength was determined using the 0.2% offset plastic strain method. The yield strength (σ_y_), ultimate tensile strength (σ_t_), fracture elongation (ε_f_), and uniform elongation (ε_u_) for each temperature were listed in Supplementary Table 2.

A HVS-1000A microhardness tester (Laizhou Huayin Test Instrument Co., Ltd.) was used to measure the microhardness of RHEA samples, with a load of 500 g and a loading time of 15 s. In Supplementary Fig. 15a, at least three data points are used for each measurement to calculate the average value.

### TEM analysis of dislocation types

The TEM analysis was conducted along the $[\bar{1}11]$ or [$1\bar{1}\bar{1}$] zone axis, and the $\vec{{{\rm{g}}}}$ vectors were determined by $\vec{{{\bf{g}}}}\,{{\boldsymbol{\cdot }}}\,\vec{{{\bf{b}}}}$ analysis to identify screw and edge dislocations (Supplementary Table 7). During the test, the zone axis may be slightly shifted to enhance contrast. When comparing the direction of the dislocation line with the Burgers vector, errors less than 5° will be disregarded. The same color is employed to signify the $\vec{{{\bf{g}}}}$ vector and its extinction dislocation lines. Due to high-temperature deformation, some dislocation lines may blur at higher-index $\vec{{{\bf{g}}}}$ vectors. After careful examination, we have labeled only the identifiable dislocation lines.

### Atomistic simulations

The local slip resistances (LSR) for edge dislocations in Nb_40_Ta_25_Ti_15_Hf_15_Zr_5_ alloy were evaluated via molecular dynamics simulations using LAMMPS software (29Aug2024 version)^66^. Interatomic interactions were modeled using the embedded-atom method (EAM) potential specific to the alloy composition^67^. A periodic array of dislocations (PAD) model was implemented within a three-dimensional periodic simulation cell by applying isotropic elastic displacement fields to all atoms. In this setup, the edge dislocation line was oriented along the *z* axis with a length of 3b or 4b, while the slip plane normal was aligned along the *y* axis. Periodic boundary conditions were enforced along the *x* and *z* directions, whereas a free surface boundary condition was applied along the *y*-direction. The LSR was computed for dislocations gliding on various slip planes, including {110}, {112}, and {123} planes. An incremental strain of 10^−5^ was applied to the simulation cell. After each strain increment, the system configuration was relaxed using the conjugate-gradient energy minimization method. The LSR was determined as the minimum stress required for the dislocation to traverse at least one lattice periodicity on the respective slip plane, following an established algorithm detailed in ref. ^29^.

### Reporting summary

Further information on research design is available in the Nature Portfolio Reporting Summary linked to this article.

## Supplementary information

Supplementary Information

Reporting Summary

Transparent Peer Review file

## Source data

Source Data

## Data Availability

All data supporting the findings in this study are available within the main text, Supplementary Information, and Source Data file. The raw data have been deposited on Zenodo: 10.5281/zenodo.18908837. Source data are provided with this paper.
