# Supplementary material for: Intrinsic edge dislocations promote high-temperature strength and ductility in additively manufactured refractory high-entropy alloys
Source: Nat Commun. 2026 Apr 24;17:5688. doi: 10.1038/s41467-026-71995-8 (PMC13319749; doi:10.1038/s41467-026-71995-8)
Supplement: Supplementary file 1 — Supplementary Information [file 41467_2026_71995_MOESM1_ESM.pdf]

Supplementary Information

for

Intrinsic edge dislocations promote high-temperature strength and ductility in additively manufactured refractory high-entropy alloys

Chunhuan Guo[1,2#], Bo Jiao[1#], Fengchun Jiang[2,3*], Wei Chen[2,3], Wenyuan Wang[1,2], Cheng Zhang[4*], Bozhao Zhang[5], Huabing Gao[1], Tao Dong[2,3], Wenyao Sun[2,3], Zubin Chen[2,3], Haixin Li[2,3], Zhenlin Yang[2,3], Shiteng Zhao[4], Jun Ding[5*] & Robert O. Ritchie[6*]

Corresponding authors: Fengchun Jiang, fengchunjiang@hrbeu.edu.cn; Cheng Zhang, bht0095@tmslab.cn; Jun Ding, dingsn@xjtu.edu.cn; Robert O. Ritchie, roritchie@lbl.gov

This file includes:

Supplementary Figs. 1 to 16

Supplementary Tables 1 to 7

Supplementary Note 1: Criteria for selecting materials

Supplementary Note 2: Optimization of LDED parameter

Supplementary Note 3: Microstructure and chemical homogeneity analysis of the additively manufactured $Nb_{40}Ta_{25}Ti_{15}Hf_{15}Zr_5$ refractory high-entropy alloy

Supplementary Note 4: Mechanical properties and strain hardening behaviors analysis

Supplementary Note 5: Estimation of dislocation density by XRD

Supplementary Note 6: EBSD analysis of multiple slip interaction mechanisms in kink bands

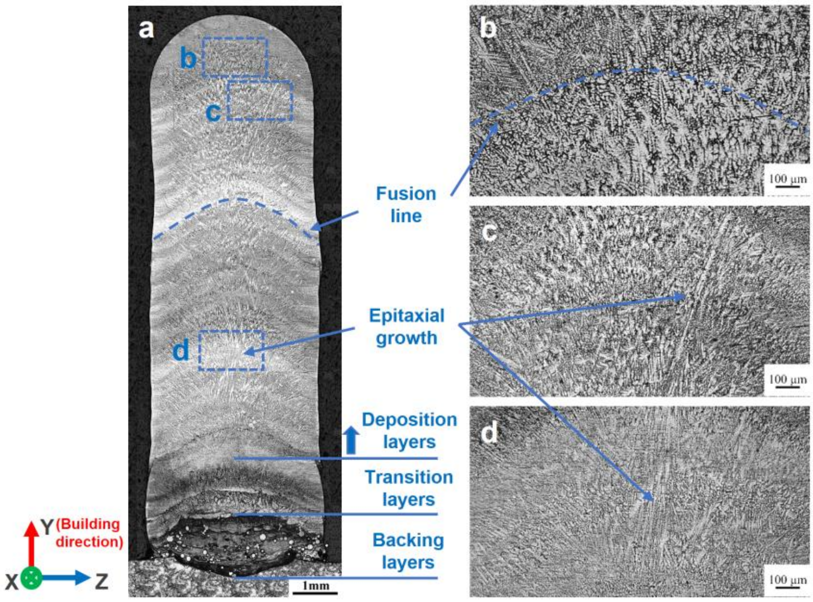

Supplementary Fig. 1 Optical microscope (OM) characterization of AM-Nb40. a, OM image of the sample (Fig. 1c) in YZ section. **b-d,** Enlarged local images at different positions in **a**.

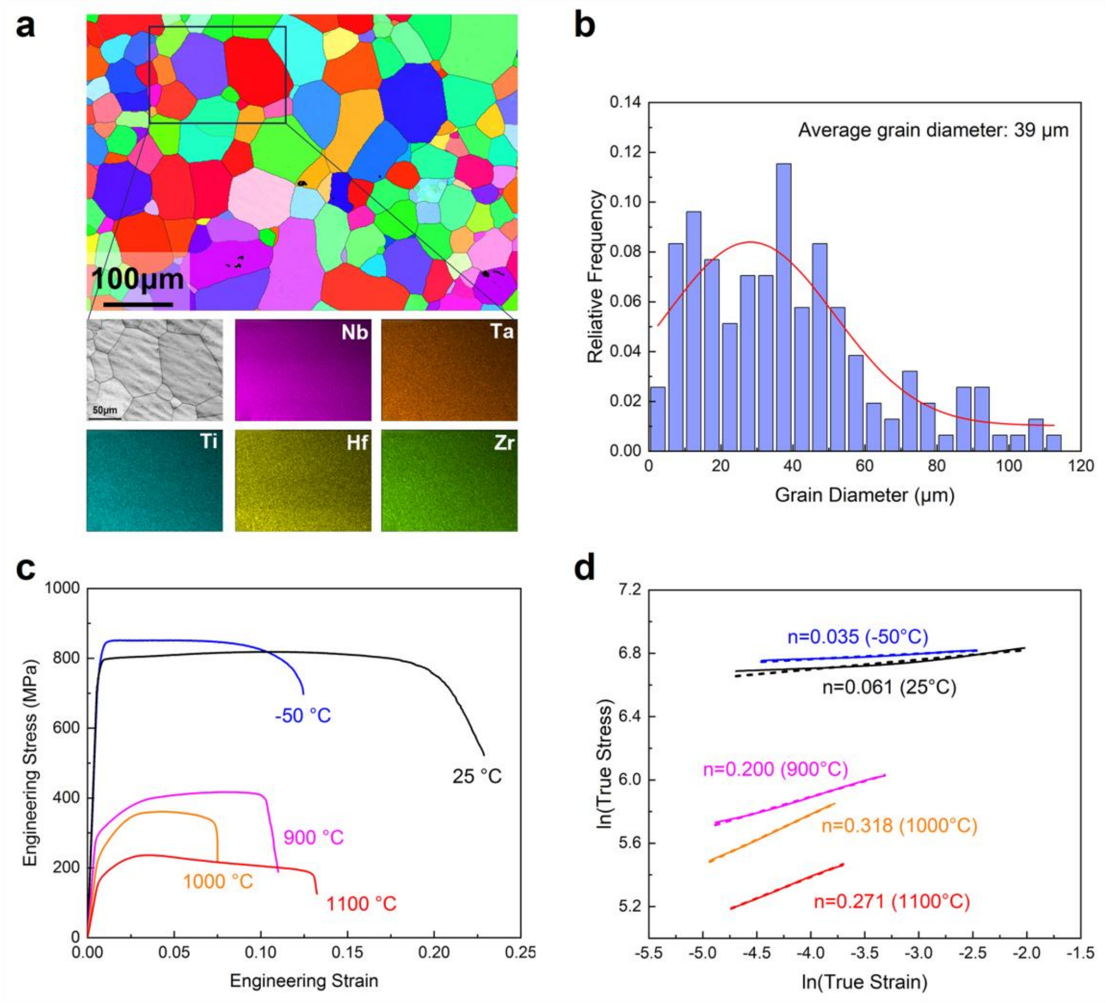

Supplementary Fig. 2 The microstructure and tensile properties of the fully recrystallized Nb40 (FR-Nb40) RHEA. a, EBSD IPF map and EDS mapping of the initial microstructure. **b,** A distribution of the grain sizes. The average grain size is 39 μm. **c,** Tensile engineering stress-strain curves measured from -50 °C to 1100 °C. The yield strength (σ_y) and tensile strength (σ_t) are marked on the curves. **d,** Strain hardening exponents obtained at each temperature. Compared to low temperature properties, the alloy has higher strain hardening exponents at elevated temperatures.

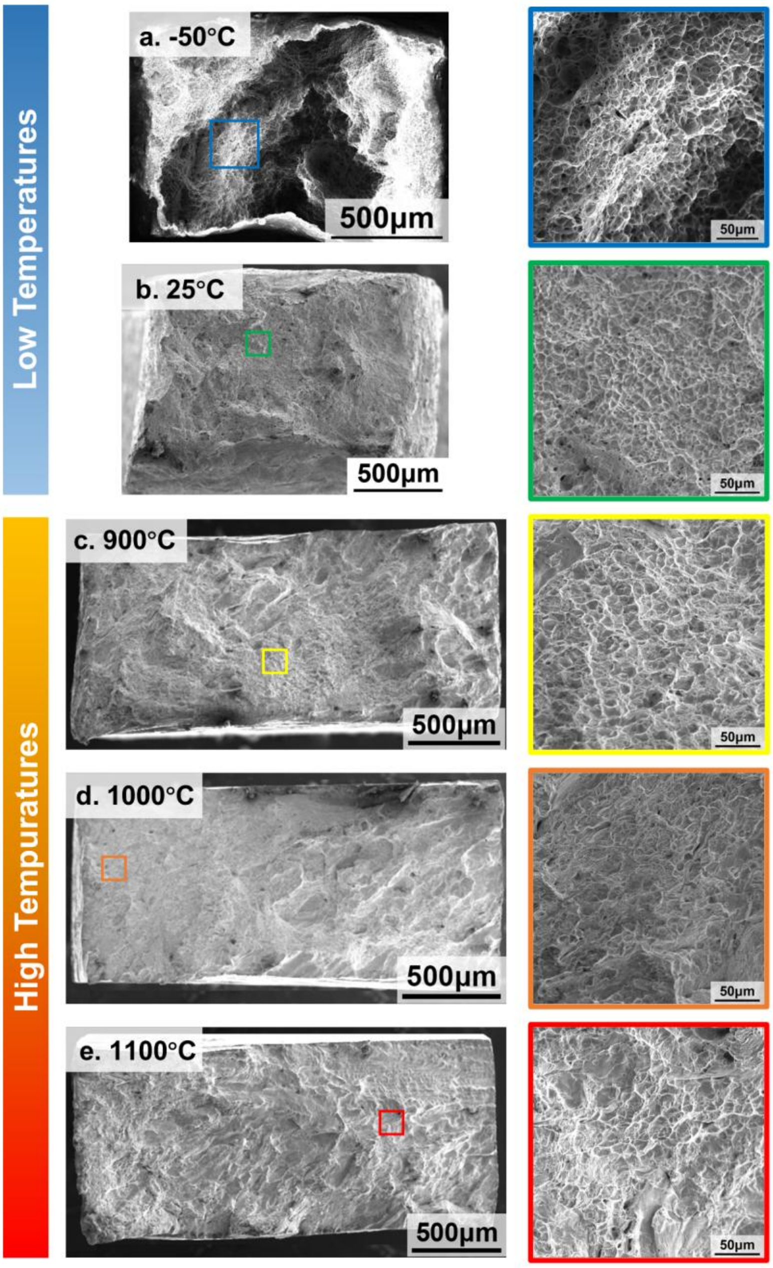

Supplementary Fig. 3 Fracture surfaces of AM-Nb40 tensile samples tested at different temperatures. a, -50 °C. **b,** 25 °C. **c,** 900 °C. **d,** 1000 °C. **e,** 1100 °C. The fracture surfaces at both low and high temperatures primarily consist of dimples (microvoid coalescence), which demonstrates a completely ductile fracture mode. The size of the dimples increases with increasing temperature. In contrast, at elevated temperatures, the fracture characteristics transition to a combination of cleavage facets and dimples, indicating the existence of intergranular fracture and revealing the transformation of fracture behavior influenced by thermal conditions. While the widespread presence of dimples confirms that ductile deformation remains dominant.

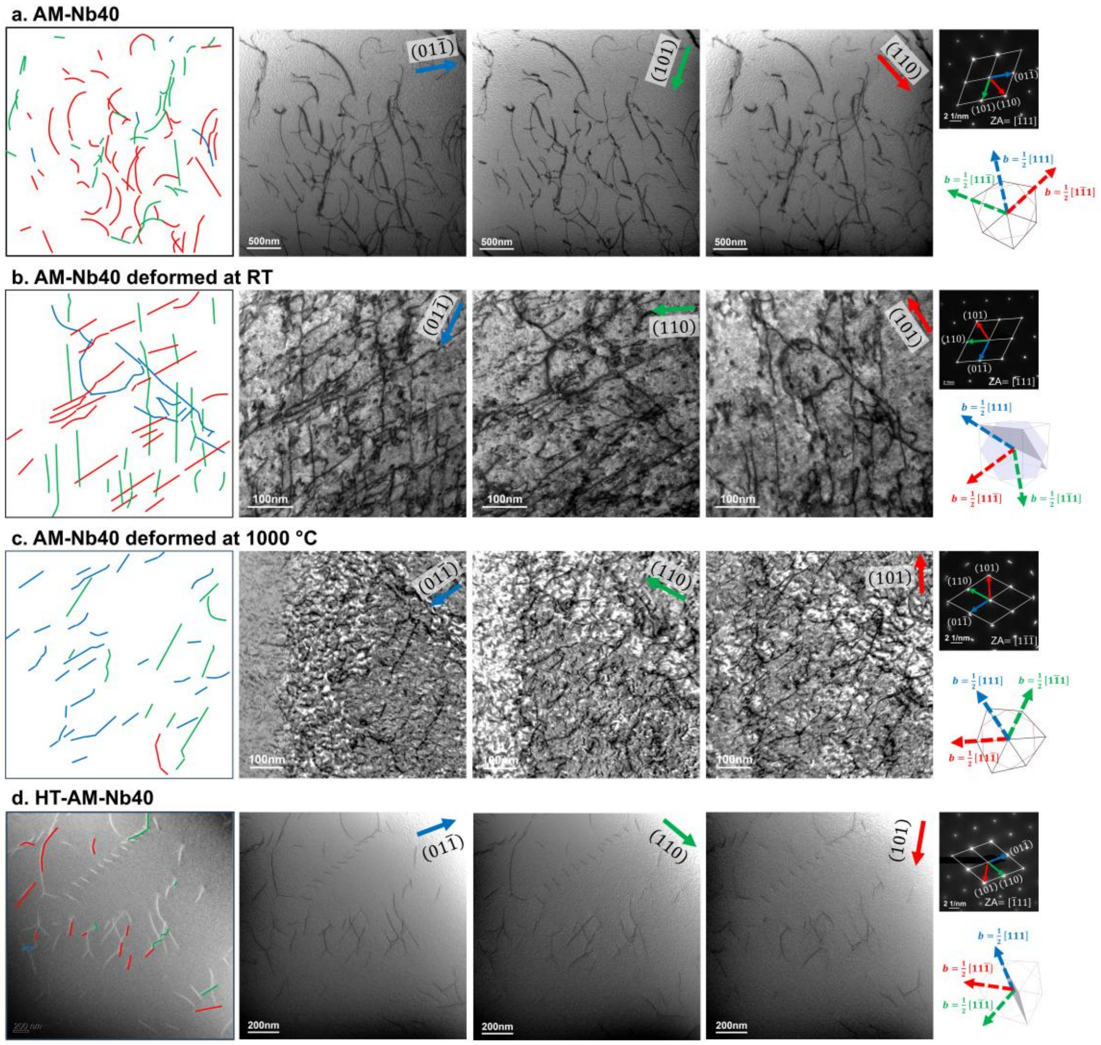

Supplementary Fig. 4 Dislocation analysis. a-d, Screw and edge dislocations were identified through g · b analysis, corresponding to Fig. 3a–d in the main text. Schematic of dislocations colored by their respective Burgers vectors. The selected area diffraction pattern (SAED) and spatial directions of Burgers vectors are marked on the right side of the images, respectively.

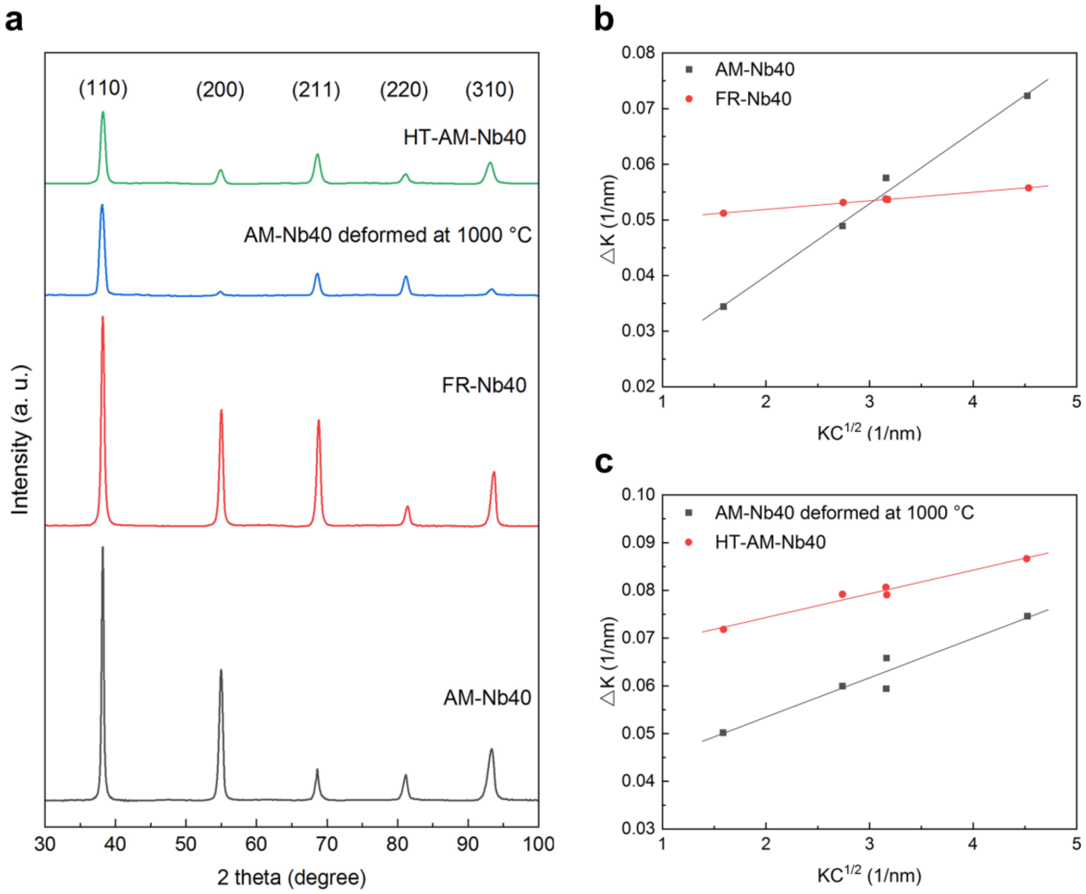

Supplementary Fig. 5 XRD analysis. a, XRD patterns. **b,c,** Modified Williamson-Hall plot.

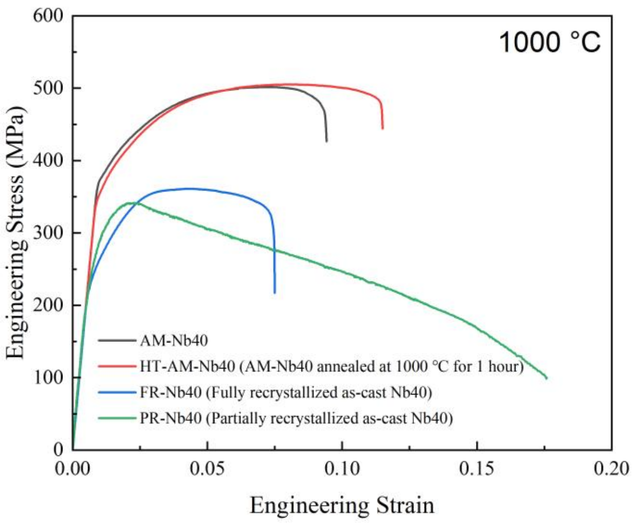

Supplementary Fig. 6 Tensile engineering stress-strain curves measured at 1000 °C of AM-Nb40, HT-AM-Nb40, FR-Nb40, and PR-Nb40.

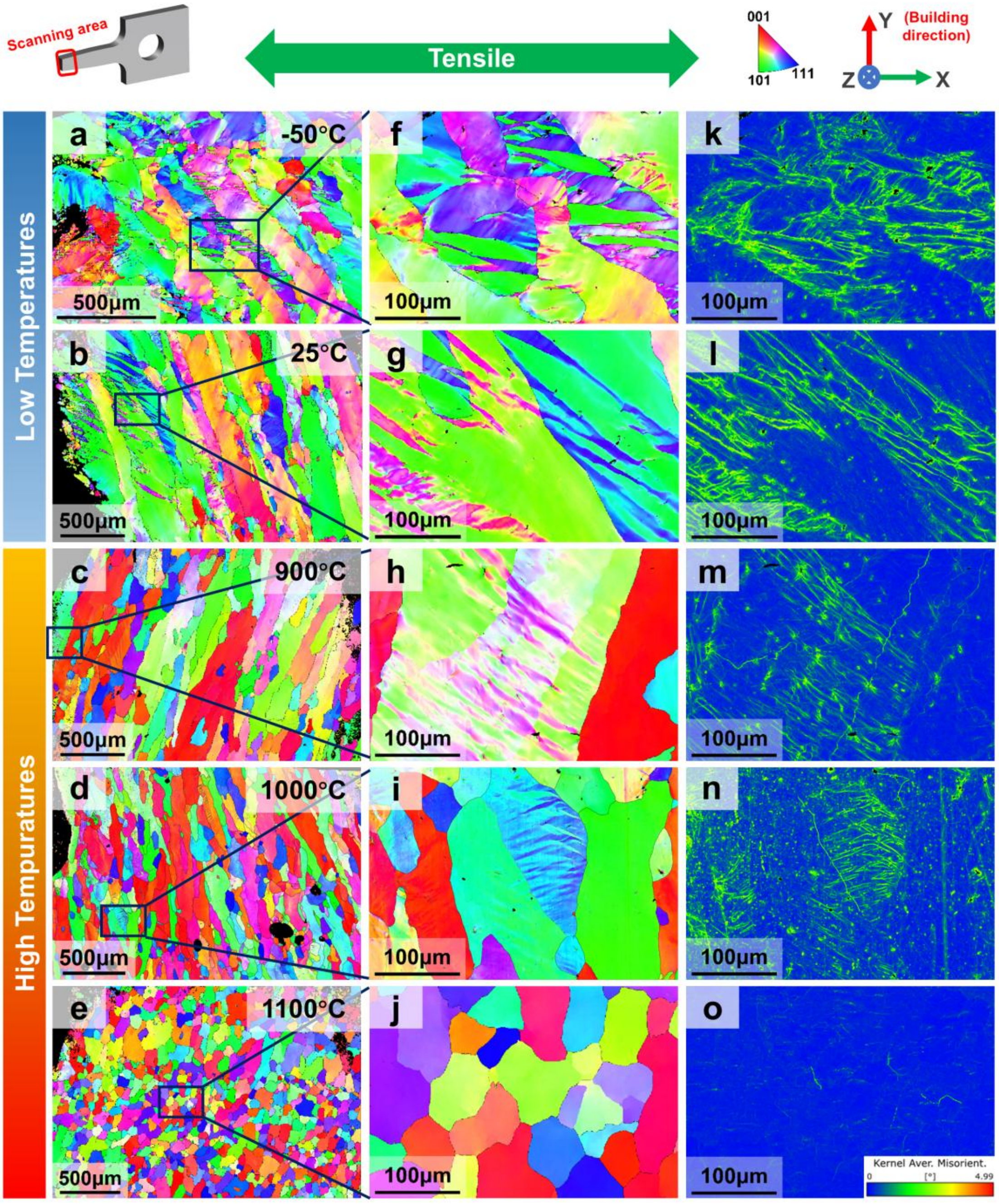

Supplementary Fig. 7 Microstructure evolution analysis via EBSD for AM-Nb40 RHEA deformed along the horizontal direction from -50 to 1100 °C. The illustration in the upper left corner shows sampling area. **a-e,** EBSD IPF maps of samples after tensile test at different temperatures. **f-j,** Local magnification images of **a-e**, respectively. After deformation at 1100 °C, a dynamic recrystallized microstructure replaced kink bands shown in **j**. **k-o** Kernel average misorientation (KAM) maps corresponding to **f-j**. Most of the geometrically necessary dislocations are confined to the boundaries of kink bands in **k-n**.

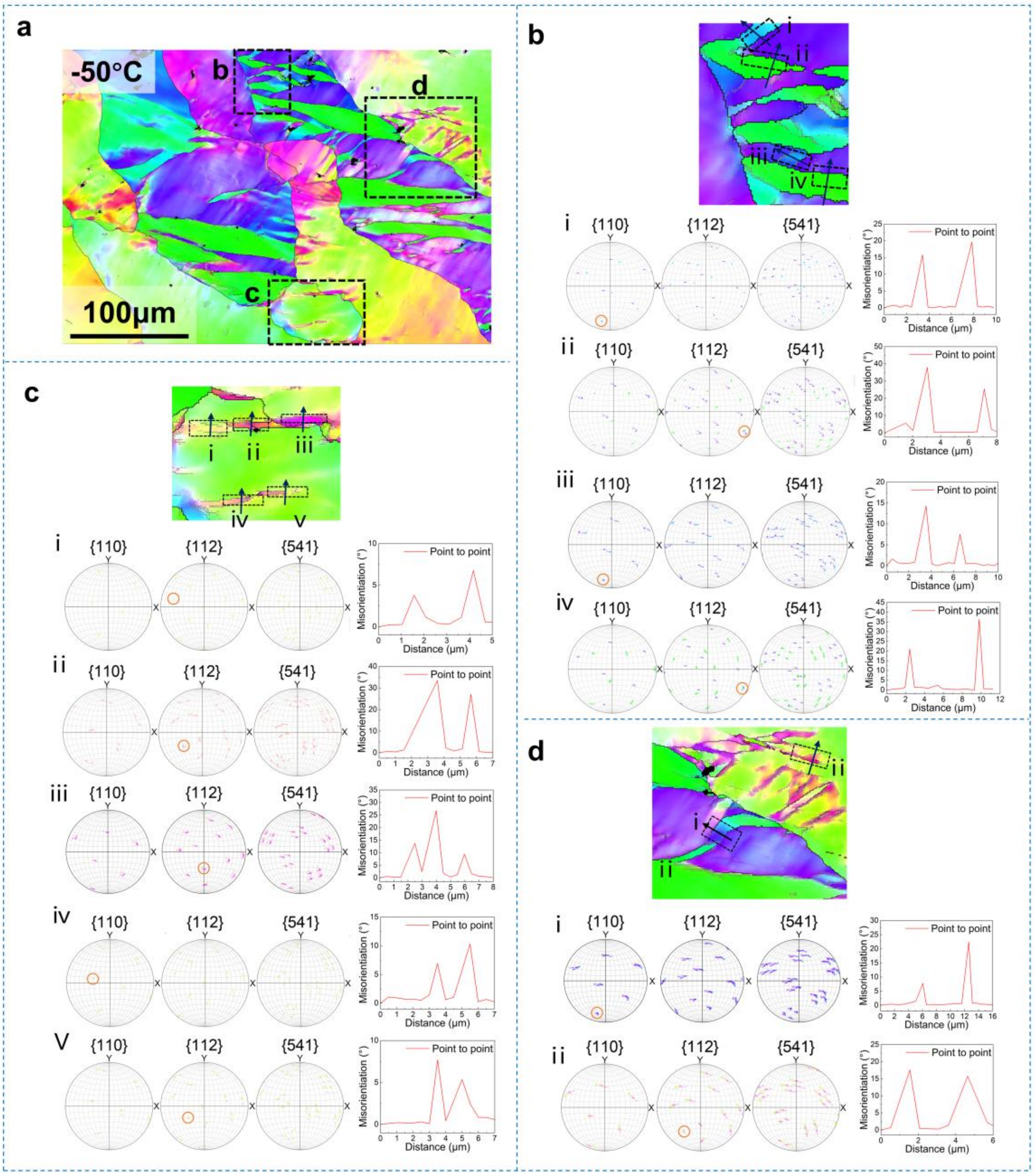

Supplementary Fig. 8 Subset pole figures and misorientation analysis of kink bands after -50 °C tensile deformation. a, The EBSD IPF+GB map of the tensile sample deformed at -50 °C (Supplementary Fig. 7f). **b-d,** Several examples of kink bands formed by tilt boundaries originating from different slip systems. The obvious polar focus in the pole figures is circled, and the slip system changes between adjacent tilt boundaries are marked by arrows.

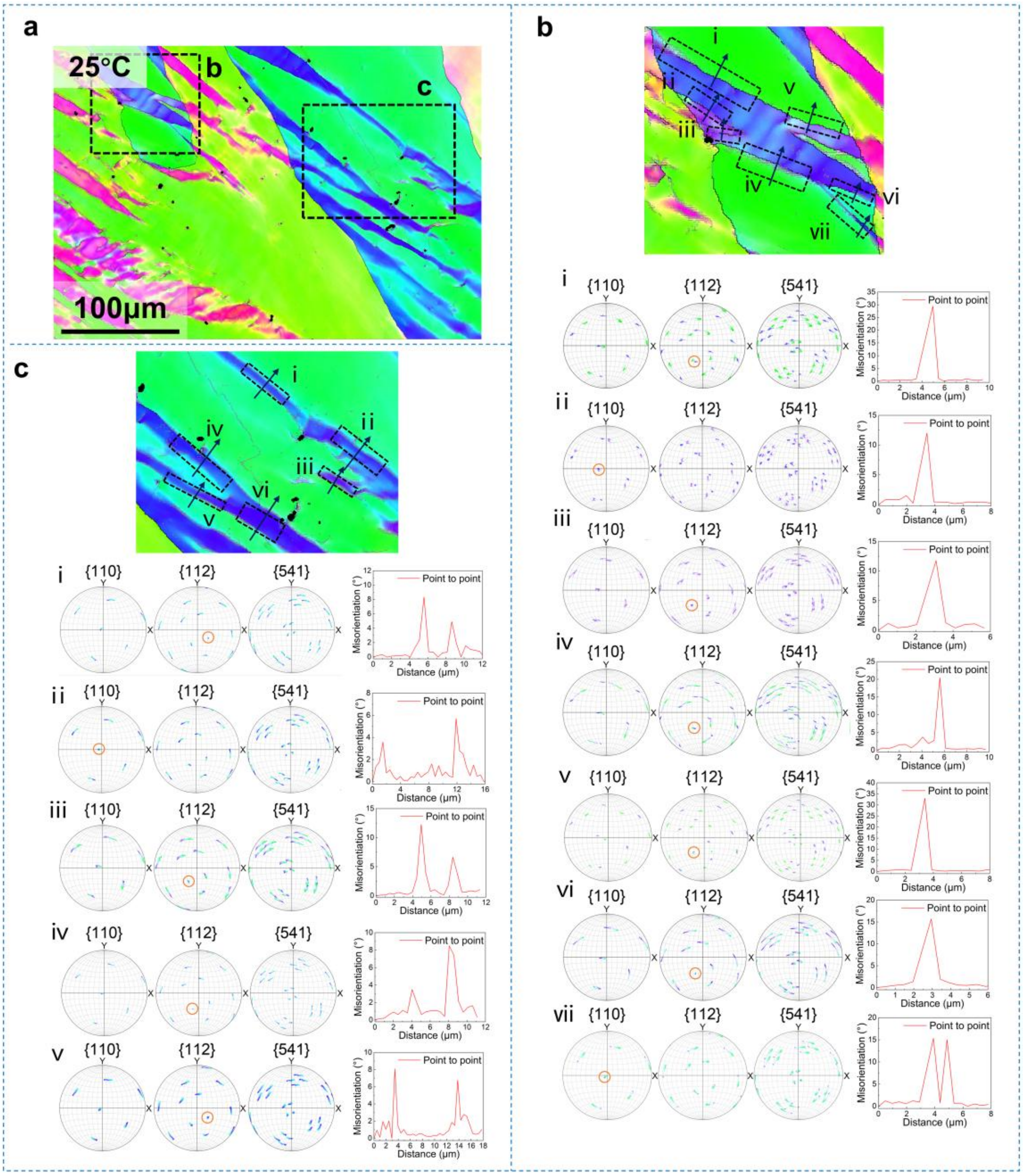

Supplementary Fig. 9 Subset pole figures and misorientation analysis of kink bands after 25 °C tensile deformation. a, The EBSD IPF+GB map of the tensile sample deformed at 25 °C (Supplementary Fig. 7g). **b,c,** Several examples of kink bands formed by tilt boundaries originating from different slip systems. The obvious polar focus in the pole figures is circled, and the slip system changes between adjacent tilt boundaries are marked by arrows.

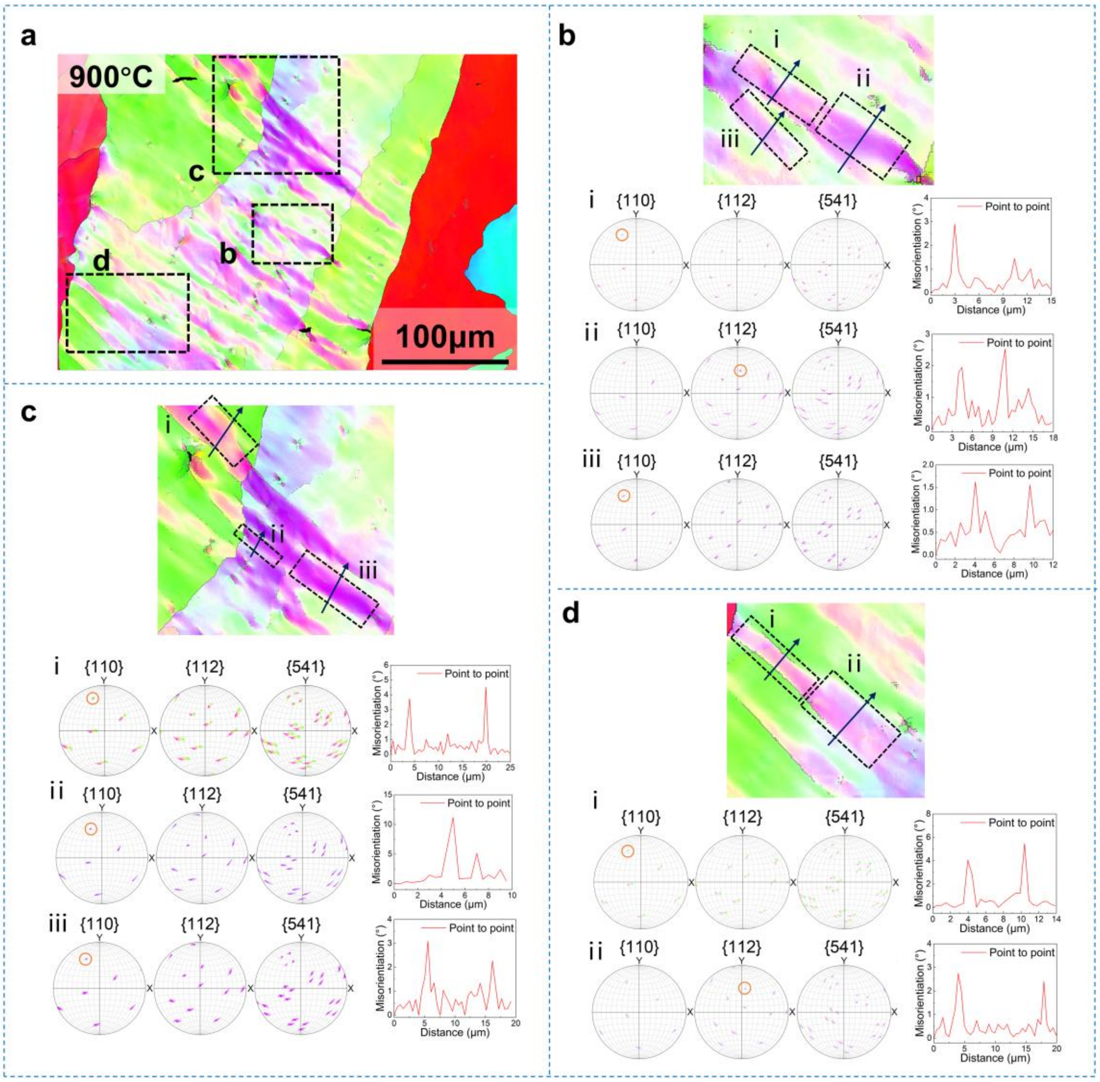

Supplementary Fig. 10 Subset pole figures and misorientation analysis of kink bands after 900 °C tensile deformation. a, The EBSD IPF+GB map of the tensile sample deformed at 900 °C (Supplementary Fig. 7h). **b-d,** Several examples of dendritic kink bands formed by tilt boundaries originating from different slip systems. The obvious polar focus in the pole figures is circled, and the slip system changes between adjacent tilt boundaries are marked by arrows.

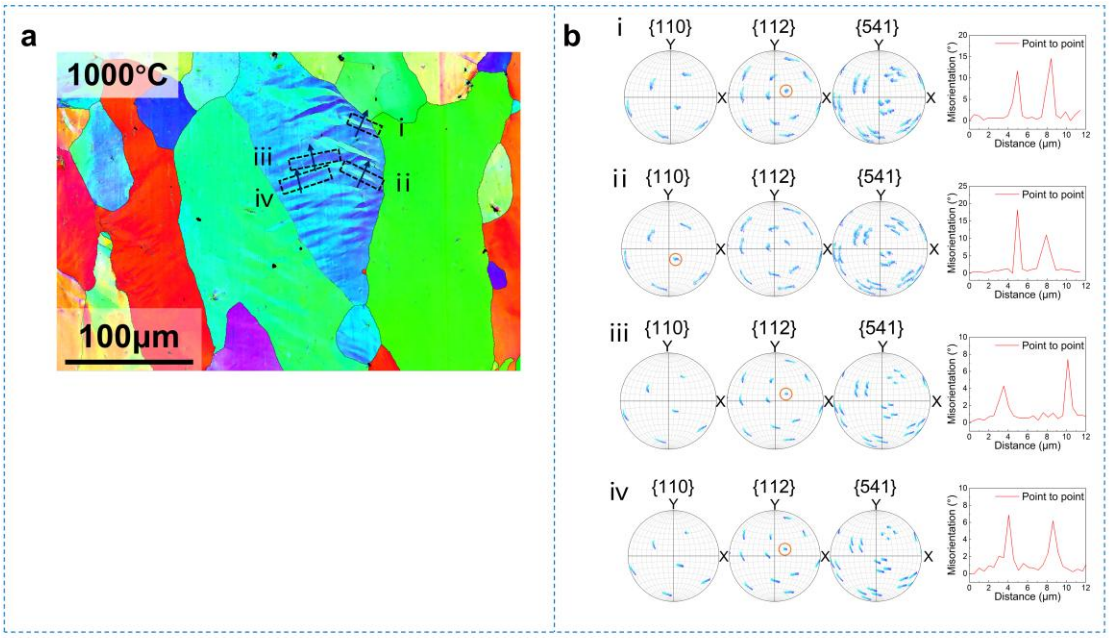

Supplementary Fig. 11 Subset pole figures and misorientation analysis of kink bands after 1000 °C tensile deformation. a, The EBSD IPF+GB map of the tensile sample deformed at 1000 °C (Supplementary Fig. 7i). **b,** Several examples of polygonal kink bands formed by tilt boundaries originating from different slip systems. The obvious polar focus in the pole figures is circled, and the slip system changes between adjacent tilt boundaries are marked by arrows.

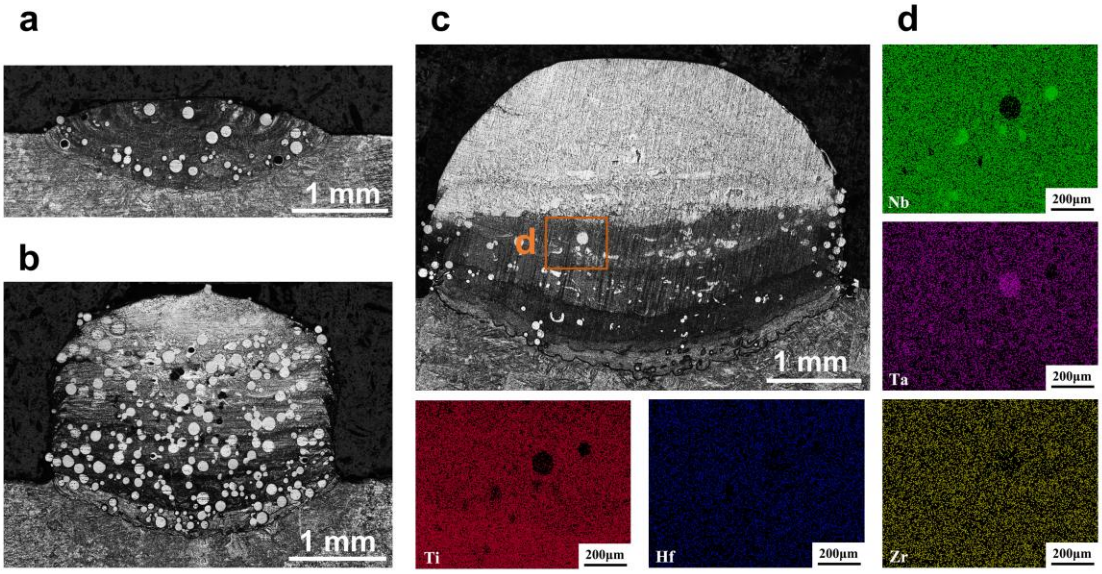

Supplementary Fig. 12 OM images and EDS mapping of sample cross-sections. a, Cross-sectional OM image of the single-layer backing structure (laser power P = 1000 W and scanning speed v = 700 mm/min, without remelting). **b,** Cross-sectional OM image of the six-layer backing structure, all samples in Supplementary Fig. 13 were validated based on this configuration. **c,** Cross-sectional OM image of sample used to determine the applicable ranges of laser power and remelting scan speed, with the corresponding process parameters listed in Supplementary Table 5. **d,** EDS mapping of the rectangle in **c**. Significant amounts of unmelted Nb and Ta powders were observed in the initial layers deposited at low power. Complete powder melting was achieved when the laser power reached 2500 W.

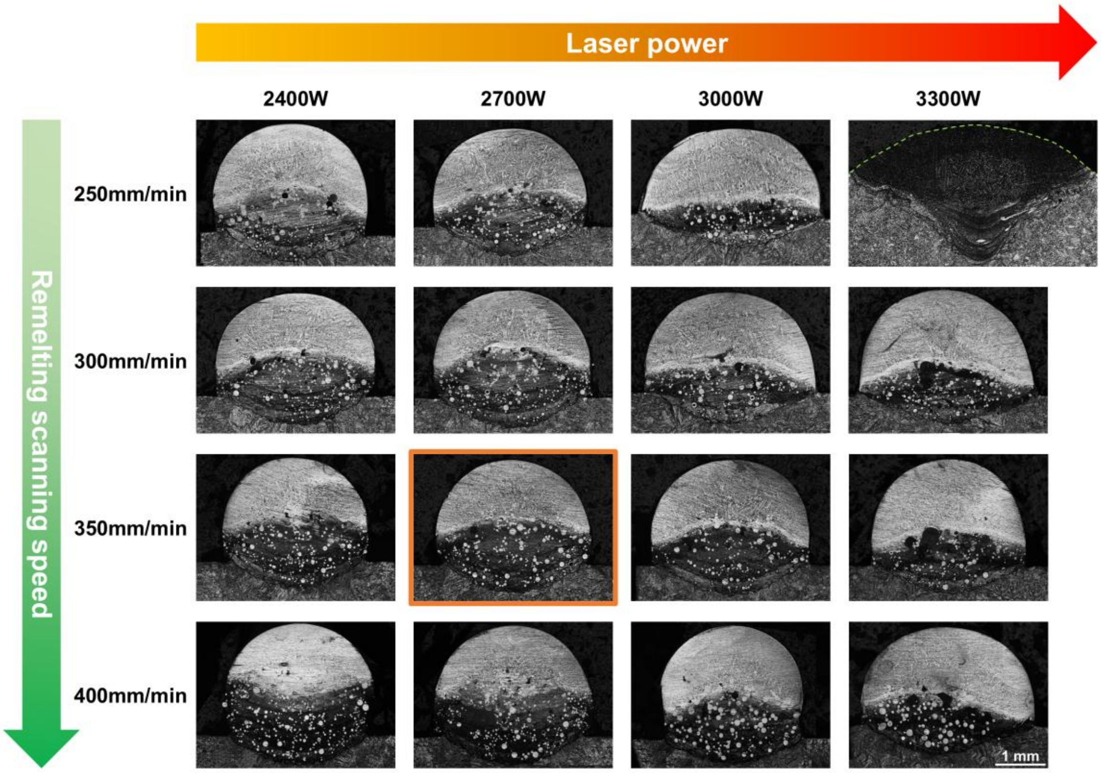

Supplementary Fig. 13 OM images of the AMed samples obtained at various process parameters. A laser power (*P*) of 2700 W combined with a remelting scanning speed (*v*) of 350 mm/min leads to the optimal forming quality, as highlighted in the figure.

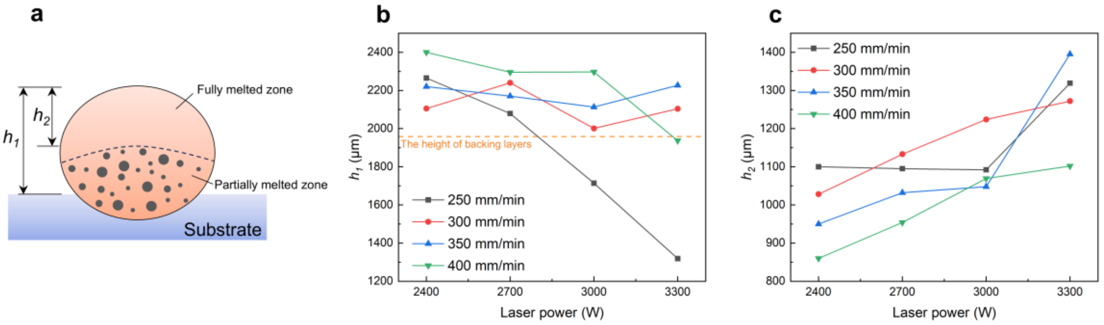

Supplementary Fig. 14 Geometric characteristics measured under varying laser powers and remelting scan speeds. a, Schematic of key geometric features in the samples. **b,c,** Variations in total sample height (h_1) and fully melted zone height (h_2) with laser power and remelting scan speed.

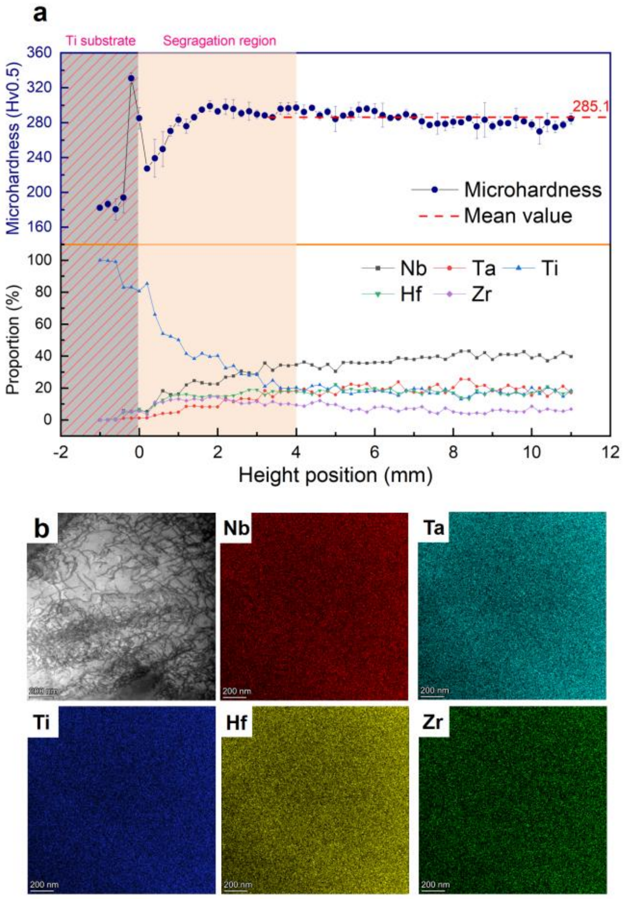

Supplementary Fig. 15 Analysis of elemental homogeneity of AM-Nb40 RHEA. a, Element proportion and microhardness of the sample (Fig. 1c) in YZ section from bottom to top. A Ti segregation region (0-4 mm in height) was observed at the base of the sample, with the microhardness and proportions stabilizing in the height range from 4 to 11 mm. The bottom 4 mm of the alloy was removed, and the remaining section was preserved for subsequent characterization and mechanical testing. **b,** TEM (BF) micrograph and corresponding EDS mapping of areas above 4 mm in AM-Nb40.

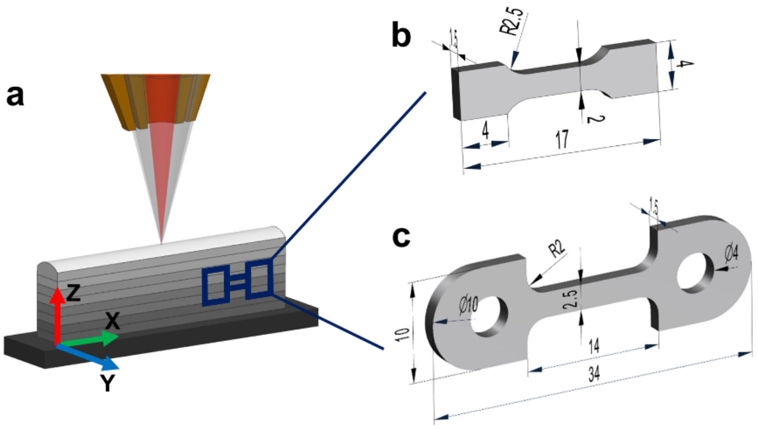

Supplementary Fig. 16 Sampling location and sample dimensions. a, Schematic illustration of a thin-wall $Nb_{40}Ta_{25}Ti_{15}Hf_{15}Zr_5$ specimen fabricated by LDED. **b,** Room temperature tensile sample configuration. **c,** Tensile sample configuration used for -50, 900, 1000, and 1100 °C.

Supplementary Table 1 The nominal chemical composition and actual chemical compositions of the AM-Nb40 and FR-Nb40 RHEAs. The actual chemical compositions (at. %) are measured by SEM-EDS, and the oxygen and nitrogen contents obtained through an Oxygen / Nitrogen detector.

Elements	Nb	Ta	Ti	Hf	Zr	O	N
Melting Point / °C	2477	3017	1668	2233	1855 ($Zr_{2.5}Nb$)		
Nominal composition / at. %	40	25	15	15	5		
AM-Nb40 / at. %	38.86	22.69	16.84	15.09	6.52	0.419	0.227
FR-Nb40 / at. %	40.63	23.51	15.95	14.63	5.28	0.268	0.061

Supplementary Table 2 A summary of tensile properties, including yield strength (σ_y), ultimate tensile strength (σ_t), fracture elongation (ε_f), and uniform elongation (ε_u) of the alloys in Fig. 2c and 2d.

Alloys	Processing Condition	Temperature (°C)	Yield strength (MPa)	Tensile strength (MPa)	Elongation to Fracture (%)	Uniform Ductility (%)	Ref.
AM-Nb40	AM	-50	814.7	898.2	16.9	6.6	This work
			820.8	874.4	16.1	5.0	
			824.4	930.7	16.6	7.0	
		25	743.6	807.3	15.3	5.9	
			767.1	818.1	14.0	5.0	
			730.3	792.6	15.0	5.6	
		900	376.9	501.3	10.3	7.7	
			385.8	528.9	9.7	7.8	
			414.8	538.9	9.5	7.2	
		1000	340.6	484.7	9.7	7.5	
			383.1	501.6	9.4	6.9	
			359.5	505.7	7.5	5.9	
		1100	284.5	396.2	8.2	7.4	
			313.1	414.0	7.3	5.3	
			317.0	417.6	5.6	4.8	
HT-AM-Nb40	AM+1000°C/1h, water quenching	1000	363.3	505.2	11.5	7.8	This work
			344.7	507.3	12.0	8.8	
FR-Nb40	Arc melting + CR85% + 1300°C/1h, water quenching	-50	820.0	852.1	12.5	4.0	This work
		25	781.3	819.0	22.9	10.5	
		900	298.6	416.8	11.0	7.7	
		1000	249.2	360.8	7.5	4.2	
		1100	175.5	236.2	13.2	3.5	
PR-Nb40 (Hetero-structure)	Arc melting + CR90% + 900 °C/1h, water quenching	25	920	1068	14.8	11.6	1
		800	451	550	10.8	3.1	
		900	398	454	11.0	2.6	
		1000	301	325	24.8	1.9	
		1100	180	253	10.8	2.9	
$Nb_{45}Ta_{25}Ti_{15}Hf_{15}$	Arc melting + CR30% + 1100°C/1h, water quenching	25	596.2	641.6	32.1	15.5	2
		800	380.0	443.7	3.2	2.3	
		950	340.8	347.0	2.2	1.1	
		1200	140.2	159.4	15.8	2.0	
NbTaTiHfZr	Wrought	25	964	~1140	16.0	-	3
		800	444	~480	2.0	-	
		1200	30	~125	121.0	-	
CM247LC	As-cast	1000	~500	-	~6.5	<2	4
GH4099	AM	1000	126	-	34.3	<1	5

AlCrCuNiFeCo	As-cast	1000	37	44	77	~2.7	6
AlCoCrFeNiTi	Cast+1220 °C/20h+900°C/5h, air cooling	1000	~130	148	92	~2.0	7
Ti-5Al-5V-2Mo-1Cr-1Fe	Wrought	950	~18	18	146	<2.0	8
Inconel 718	Hot-Rolled Round + Annealed and Aged	25	1124	1365	21	-	9
		538	1020	1193	16	-	
		649	965	1103	15	-	
		760	800	852	5	-	
		871	~309	~309	~64	-	
		982	~140	~140	~171	-	
		1093	~77	~77	~138	-	
HAYNES Waspaloy	Wrought	25	899	1304	24.5	-	10
		538	812	1175	22.0	-	
		649	784	1137	31.9	-	
		760	706	822	32.8	-	
		815.5	517	633	39.7	-	
		871	357	456	48.0	-	
		927	210	297	57.7	-	
		982	132	174	57.8	-	
		1093	31	51	135.5	-	
Mar-M247	Cast + Heat treatment	25	859.8	988.7	5.9	-	11
		538	811.5	1006.6	7.9	-	
		649	783.2	983.2	6.9	-	
		760	808.1	1000.4	4.3	-	
		871	704.6	852.9	3.2	-	
		982	422.0	570.2	6.4	-	
		1038	297.9	407.5	8.3	-	
CMSX-4	Cast + Heat-Treated Condition A	24	888	894	22.0	-	12
		650	907	1031	13.8	-	
		800	916	1151	22.1	-	
		1094	321	459	28.8	-	

Supplementary Table 3 The dislocation density for different samples.

Sample	ρ ($\times 10^{13}$ m^{-2})
AM-Nb40	6.26 ± 0.808
FR-Nb40	0.0893 ± 0.055
AM-Nb40 deformed at 1000 °C	2.53 ± 0.774
HT-AM-Nb40	0.914 ± 0.180

Supplementary Table 4 Physical property data of raw powder.

Powder feedstock	Particle size (μm)	D10 (μm)	D50 (μm)	D90 (μm)	Tap density (g/cm^3)	Apparent density (g/cm^3)	Flowability (s/50g)
Nb	53-150	68.1	105.2	146.7	5.43	4.21	11.50
Ta	53-150	53.3	89.2	141.2	10.28	9.87	5.49
Ti	53-150	60.1	87.0	126	2.70	2.40	30.31
Hf	53-150	70.7	91.4	121.4	8.39	7.95	7.63
$Zr_{2.5}Nb$	53-150	62.2	92.9	137.3	4.15	3.83	19.7
Powder blends	53-150	61. 7	94.3	135.6	6.42	5.66	12.65

Supplementary Table 5 Deposition parameters of the sample in Supplementary Fig. 12c.

Layer number	Power (W)	Scanning speed (mm/min)	Powder feed rate (r/min)
1	1000	700	0.4
Remelting	1000	350	0
2	1300	700	0.4
Remelting	1300	350	0
3	1600	700	0.4
Remelting	1600	350	0
4	1900	700	0.4
Remelting	1900	350	0
5	2200	700	0.4
Remelting	2200	350	0
6	2500	700	0.4
Remelting	2500	350	0

Supplementary Table 6 The LDED strategy and related parameters used in this study.

Layer	Layer number	Power (W)	Scanning speed (mm/min)	Powder feed rate (r/min)
Backing layers	1-6	1000	700	0.4
Transition layers	7	1500	700	0.4
	Remelting	1500	350	0
	8	2000	700	0.4
	Remelting	2000	350	0
	9	2500	700	0.4
	Remelting	2500	350	0
Deposition layers	10	2700	700	0.4
	Remelting	2700	350	0
	Repeat the 10th layer and its remelting process to the required height.			

Supplementary Table 7 The analysis of $\vec{g} \cdot \vec{b}$ under $[\bar{1}11]$ and $[1\bar{1}\bar{1}]$ zone axes.

Zone axis	g vector	Burgers vector			
		±1/2 [111]	±1/2 $[\bar{1}11]$	±1/2 $[1\bar{1}1]$	±1/2 $[11\bar{1}]$
$[\bar{1}11]/[1\bar{1}\bar{1}]$	(110)	2	0	0	2
	$(01\bar{1})$	0	0	-2	2
	(101)	2	0	2	0

Supplementary Note 1: Criteria for selecting materials

The non-equiatomic $Nb_{40}Ta_{25}Ti_{15}Hf_{15}Zr_5$ (at. %, abbreviated as Nb40) RHEA is derived from the $Nb_{60}Ta_{25}Ti_{15}$ matrix. The addition of Hf and Zr not only enhances ductility but also improves strength by increasing lattice distortion[13-15]. Remarkably, Nb40 can be cold-rolled from the as-cast state to a reduction exceeding 90% without surface treatments or intermediate annealing, which exhibits outstanding strength and ductility over a broad temperature range (-196 to 1100 °C)[1]. The exceptional cold workability originates from the activation of high density dislocations and deformation twins, and is facilitated by extensive diffusion pathways that enable homogenization at reduced annealing temperatures and shortened durations[16]. Similarly, the additive manufacturing (AM) process of Nb40 generates high-density intrinsic dislocations, and therefore endows the as-printed alloy with comparable cold workability, which is a distinctive advantage absent in most AMed RHEAs[17-19]. Furthermore, defect-free bulk RHEAs with superior tensile plasticity, including NbTiHfZr[20], $Ti_{42}Hf_{21}Nb_{21}V_{16}$[21,22], and $Ti_{40}Hf_{10}Nb_{12}V_{38}$[23] have been successfully fabricated via elemental powder-based AM. However, no studies have yet reported tensile ductility in Ta-containing AMed RHEAs[24-26]. Due to their extremely high melting points, full-scale Ta-containing RHEAs are challenging to fabricate via elemental powder-based AM, which restricts mechanical property measurements to compression testing[27-30]. In order to fill the gap, Nb40 with exceptional deformability was chosen as the model alloy in this study.

Supplementary Note 2: Optimization of LDED parameter

Laser directed energy deposition (LDED) is a layer-by-layer process. The process parameters have an important influence on the microstructure and quality of fabricated samples[20]. In traditional additive manufacturing of low melting point alloys[31], systematic analysis of single-track morphology and forming quality is essential to optimize parameter compatibility and achieve high-quality deposition layers. In this study, the process window for single-track deposition involves Ta powder with an ultrahigh melting point (3017°C), requiring extreme heat input for consolidation. However, excessive power can induce defects such as burn-through and porosity in Ti substrates, severely compromising print quality. To address this, we employed a backing layer to ensure root formation and introduced laser remelting to enhance high melting point powder fusion. Single-track experiments on identical backing layers (Supplementary Fig. 12b) were conducted to determine the optimal process window, with laser power (P) and remelting scan speed (v) as key variables[20,32,33].

We initially determined the value ranges of two key variables by progressively increasing the laser power (P) layer-by-layer (Supplementary Fig. 12c), with the corresponding process parameters listed in Supplementary Table 5. At a remelting scan speed of 350 mm/min, complete melting of the feedstock powder was achieved when the laser power reached 2500 W. Consequently, the laser power (P) range of 2400-3300 W and remelting scan speed (v) range of 250-400 mm/min were selected for printing parameter optimization. Optical micrographs of sample cross-sections are presented in Supplementary Fig. 13. The key geometric features are defined in Supplementary Fig.

14a. The total sample height (h_1) and fully melted zone height (h_2) of the deposited samples are summarized in Supplementary Fig. 14b and 14c, respectively. At relatively high power levels (3000-3300 W), excessive melt pool turbulence resulted in substantial widening of the molten zone. Conversely, insufficient energy density leading to melt pool instability tended to produce spherical pores. As highlighted in Supplementary Fig. 13, the optimal processing window was identified as P = 2700 W with v = 350 mm/min with the energy density 115.7 J/mm^3, the detailed process parameters are shown in Supplementary Table 6. The energy density (E) is obtained by the following formula:

$$E = \frac{P}{vht} \tag{1}$$

where v is scanning speed, h is hatch spacing, and t is layer thickness.

Supplementary Note 3: Microstructure and chemical homogeneity analysis of the additively manufactured $Nb_{40}Ta_{25}Ti_{15}Hf_{15}Zr_5$ refractory high-entropy alloy

As shown in Supplementary Fig. 1, the AMed $Nb_{40}Ta_{25}Ti_{15}Hf_{15}Zr_5$ (AM-Nb40) exhibits a characteristic dendritic and interdendritic microstructure under the optical microscopy (OM), which is a manifestation of the differential melting points of its constituent elements[34]. Due to the cyclic melting process of LDED, dendritic crystals epitaxially grow through fish-scale-like fusion lines[35]. The frequency of thermal cycling decreases with increasing layers, and the dendrites gradually shorten (Supplementary Fig. 1b-d). Figure 1e shows a coarse columnar grain morphology along the building direction (BD) with some small partially recrystallized grains. This is quite different from the equiaxed grains formed in $Nb_xHfTiZr$ RHEA produced via elements-powder-based LDED[20]. The differing grain morphology is attributed to the increased heat input needed for the melting of Ta in the raw materials, leading to a higher ratio between temperature gradient (G) at the solid/liquid interface and solidification rate (R)[36].

As shown in the TEM bright-field (BF) image in Fig. 1g, the AM-Nb40 sample contains a high density of intrinsic dislocation, which is a characteristic feature resulting from the AM process. According to previous studies[37-40], these intrinsic dislocations typically initially form during rapid solidification and subsequently accommodate thermal deformation through strain localization under cyclic heating. This process closely mirrors repeated tension-compression fatigue deformation and represents the most significant mechanism for dislocation accumulation in AM alloys[41]. This has consequently led to the high initial dislocation density observed in our study. Detailed

estimation of dislocation density is provided in Supplementary Note 5.

The unmelted powders and various levels of etching are clearly visible at the sample's root (Supplementary Fig. 1a). The unmelted powder results from the low output power in backing layers. Furthermore, sustained high thermal input induces severe Ti segregation. To quantitatively characterize elemental segregation, we performed energy-dispersive X-ray spectroscopy (EDS) point scanning and microhardness analysis at 0.2 mm intervals from the bottom to the top on the sample longitudinal section in Supplementary Fig. 15a. A distinct Ti segregation region is observed at the bottom 4 mm of the sample, with the composition and microhardness stabilizing beyond 4 mm. Samples were collected from the 4-11 mm height area of the sample and EDS surface scanning was conducted at various magnifications. The low-magnification SEM-EDS results confirm the presence of elemental segregation at this micro-scale (Fig. 1f). The dendrite regions are rich in Ta, obviously due to the higher melting point, and the inter-dendritic regions are rich in Ti and Zr because of their low melting points. However, it reveal that AMed RHEAs exhibit significantly reduced segregation extent compared to conventionally as-cast RHEAs, which notably documented in prior studies[24,42]. The LDED process involves non-equilibrium solidification with extremely high cooling rates, which significantly reduces atomic diffusion time and consequently suppresses elemental segregation. The high-magnification EDS results from TEM indicated the absence of elemental segregation in AM-Nb40 (Supplementary Fig. 15b).

The actual chemical compositions from EDS are approximately identical to the

nominal compositions (Supplementary Table 1), demonstrating that the exceedingly high energy input during the LDED process did not induce evaporation of low melting-point elements. This finding substantiates the reliability of the optimized element-powder-based LDED parameters and confirms their efficacy in preserving the intended elemental ratios throughout the fabrication process. All AM-Nb40 samples have their bottom 4 mm removed, and subsequent testing is conducted on the remaining near-homogeneous section.

Supplementary Note 4: Mechanical properties and strain hardening behaviors analysis

For comparison purposes, the fully recrystallized as-cast Nb40 (FR-Nb40) samples with low intrinsic dislocation density were fabricated by cold rolling and annealing (detailed in Methods). The FR-Nb40 exhibits a single-phase BCC microstructure with equiaxed grains averaging 39 μm in size (Supplementary Fig. 2a and 2b). The engineering stress-strain curve of the FR-Nb40 (Supplementary Fig. 2c) reveals a yield strength (σ_y) of ~781.3 MPa and an elongation to fracture of ~22.9% at room temperature, both strength and elongation are higher than AM-Nb40. With increasing temperature, the σ_y values are ~298.6 MPa (at 900°C), ~249.2 MPa (at 1000°C), and ~175.5 MPa (at 1100°C). These values are significantly lower than those of the AM-Nb40.

Supplementary Fig. 2d shows that the strain hardening exponents (*n*) increases from 0.035(-50°C) to 0.200 (900°C), 0.318 (1000°C), and 0.271 (1100°C) with rising temperature. This trend is consistent with that observed in AM-Nb40 (Fig. 2b) and $Nb_{45}Ta_{25}Ti_{15}Hf_{15}$[2] RHEAs. This indicates that these cognate RHEAs share the same dislocation-dominated deformation mechanism at investigated temperature range. Specifically, the deformation is dominated by screw dislocations at room temperature and by edge dislocations at high temperatures. At room temperature, the high density of intrinsic screw dislocations in AMed RHEA does not provide significant strengthening. The extra dislocation pinning effects and the formation of a high density of intersecting kinks in BCC RHEAs[43-45]. The high density of cross-kinks is detrimental

to uniform and stable plastic deformation, thereby accelerating the development of stress concentrations[46]. Conversely, the FR-Nb40 with finer grains and lower initial dislocation density exhibits a superior strength-ductility combination.

In prior studies[47,48], substantial deformation twins were observed following cryogenic deformation, where the TWIP (twinning-induced plasticity) effect imparted peak ductility. However, at -50 °C, no twins formed in AM-Nb40 (Supplementary Fig. 7a), and dislocation slip remained the dominant mechanism. The cryogenic environment reduced thermal activation probability, resulting in insufficient dislocation storage in FR-Nb40 with low intrinsic dislocation density. Infrequent dislocation interactions led to weak strain-hardening capability, preventing sustained plastic flow. Consequently, early necking occurred, yielding a uniform elongation of merely 2.9%. This phenomenon aligns with findings reported for $Ti_{48.9}Zr_{32.0}Nb_{12.6}Ta_{6.5}$ RMEA under -88 °C tensile testing[49]. In contrast, for AM-Nb40 with high dislocation density, cryogenic conditions suppressed screw dislocation cross-slip, enforcing more planar slip across fewer glide systems. This simultaneously enhanced yield strength (requiring higher stress to mobilize dislocations) and promoted dislocation multiplication and storage. The resulting planar slip homogenized plastic deformation distribution, delaying necking. Ultimately, AM-Nb40 achieved superior post-elongation compared to its room-temperature performance.

At high temperatures, the edge dislocation-dominated strengthening mechanism is no longer limited by kink nucleation. The intrinsic edge dislocations promote strain hardening and uniform plastic deformation through their high slip resistance and

multiplication mechanisms. Concurrently, kink bands optimize dislocation density and distribution, effectively regulating strain gradients. Dislocations multiplication and kink band nucleation occur simultaneously, and it is to ensure that the sufficient strain hardening and compatibility stabilize uniform plastic flow and prevent stress localization. The synergistic mechanism between strain hardening and stress relaxation at elevated temperatures confers AM-Nb40 with superior uniform elongation and tensile strength compared to the FR-Nb40 (Fig. 2c). And, the strength of AM-Nb40 shows no significant reduction after the dislocation annihilation induced by the 1 h heat treatment (HT-AM-Nb40). As shown in Supplementary Fig. 6, these values remain notably higher than those of the fully recrystallized (FR-Nb40), partially recrystallized (PR-Nb40)[1] states, while demonstrating a clear improvement in elongation compared to the AM-Nb40. Clearly, the dislocations formed during the AM process remain stable even after a period of high-temperature exposure and continue to provide a high-temperature strengthening effect.

Supplementary Note 5: Estimation of dislocation density by XRD

Based on the XRD pattern shown in Supplementary Fig. 5a, the dislocation density of the AM-Nb40, AM-Nb40 deformed at 1000 °C, HT-AM-Nb40, and FR-Nb40 were estimated using the modified Williamson-Hall method[50], and it can be expressed as:

$$\Delta K \cong \frac{0.9}{D} + \left(\frac{\pi M^2 b^2}{2}\rho\right)^{\frac{1}{2}} \left(K\bar{C}^{\frac{1}{2}}\right) + O\left(K^2\bar{C}^{\frac{1}{2}}\right) \tag{2}$$

$$\Delta K = cos\theta * \beta / \lambda \tag{3}$$

$$K = 2sin\theta / \lambda \tag{4}$$

where ΔK is the strain broadened full width at half maximum (FWHM) in reciprocal space given by Eq. (3), and θ, β and λ are the Bragg diffraction angle, FWHM for the {hkl} peak, and wavelength of CuKα radiation (1.5406 Å), respectively. D represents the average grain size, b represents Burgers vector (~0.2927 nm), and ρ represents the dislocation density and M represents the dislocation arrangement parameter. K represents the modulus of the diffraction vector given by Eq. (4). For polycrystalline materials containing dislocations, the linear fitting between ΔK and $K\bar{C}^{\frac{1}{2}}$ is obtained from the modified Williamson-Hall plot, and the first-order coefficient after fitting is $\left(\frac{\pi M^2 b^2}{2}\rho\right)^{\frac{1}{2}}$. Where $\bar{C}$ is the average contrast factor, and its formula is as follows:

$$\bar{C}_{hkl} = \bar{C}_{h00}\left[1 - q\frac{h^2k^2 + h^2l^2 + k^2l^2}{(h^2 + k^2 + l^2)^2}\right] \tag{5}$$

In equation (5), hkl are the reflection indexes and $\bar{C}_{h00}$ is the contrast factor. Referring to previous work[51], the value of $\bar{C}_{h00}$ is between 0.305 ± 0.015 and 0.260 ± 0.045, and the average value of 0.28 is used in the fitting process to determine the dislocation density. The value of q is selected as 2.0[50,51]. The modified Williamson-Hall fitting

curves of different samples are shown in Supplementary Fig. 5b and 5c. The dislocation density values of different samples are given in Supplementary Table 3.

Among them, the dislocation density of AM-Nb40 was measured at 6.26×10^{13} m^{-2}. However, the modified Williamson-Hall analysis involves assumptions and parameters that can introduce uncertainty. To provide context, we referred to the reported dislocation density values for various AM refractory metals and alloys[38,52-55]. These studies employed Electron Channeling Contrast Imaging (ECCI)[38], Kernel Average Misorientation (KAM)[52-54], and the Williamson-Hall (WH)[55] methods to analyze dislocation density, with the results consistently around 10^{13} m^{-2}. Our evaluated dislocation density value for AM-Nb40 is consistent with this reported range and is corroborated by KAM and TEM characterization techniques. Based on unified parameters, our dislocation density assessment is mainly correct and reliable. These values primarily reveal relative trends in dislocation density under different states rather than absolute values.

Supplementary Note 6: EBSD analysis of multiple slip interaction mechanisms in kink bands

Kink bands are formed by the tilt boundaries caused by edge dislocation arrays. The Taylor axes of tilt boundaries can be determined through subset pole diagram analysis. Multiple pole groups are formed in each pole figure, with the apparent pole focus projected from the unique common lattice plane of parent grain and kink band on both sides of the tilt boundary, which is the normal plane of the Taylor axis. Based on this analysis, the {110}<111>, and {112}<111> slip systems are all activated during the formation process of the identified kink bands in AM-Nb40. Supplementary Figs. 8-11 highlight potential slip activation across corresponding kink bands marked by circles and emphasize the involvement of multiple slip systems during kink band development. Notably, kink bands can result from the formation of tilt boundaries across several slip systems. The edge dislocation arrays are activated from high dislocation density regions, mostly at grain boundaries (Supplementary Figs. 8-11). A continuous stream of dislocations moves along the slip plane, with intersection and accumulation. This process can simultaneously induce the formation of one or more kink bands. Under the complex stress at the crack tip, the crystal orientation rotates to adapt to local strain. This leads to flexible orientation changes in the development process of kink bands.

References for Supplementary Information

1 Zhang, C. *et al.* Strong and ductile refractory high-entropy alloys with super formability. *Acta Mater.* **245**, 118602 (2023).

2 Cook, D. H. *et al.* Kink bands promote exceptional fracture resistance in a NbTaTiHf refractory medium-entropy alloy. *Science* **384**, 178-184 (2024).

3 Mills, L. H. *et al.* Temperature-dependent tensile behavior of the HfNbTaTiZr multi-principal element alloy. *Acta Mater.* **245**, 118618 (2023).

4 Ghoussoub, J. N. *et al.* A new class of alumina-forming superalloy for 3D printing. *Addit. Manuf.* **52**, 102608 (2022).

5 Nie, Z., Guo, Q., Zhao, Y., Ma, Z. & Liu, Y. Temperature dependence tensile behaviors of additively manufactured GH4099 Ni-based superalloy. *Mater. Sci. Eng. A* **899**, 146464 (2024).

6 Kuznetsov, A. V., Shaysultanov, D. G., Stepanov, N. D., Salishchev, G. A. & Senkov, O. N. Tensile properties of an AlCrCuNiFeCo high-entropy alloy in as-cast and wrought conditions. *Mater. Sci. Eng. A* **533**, 107-118 (2012).

7 Daoud, H. M., Manzoni, A. M., Wanderka, N. & Glatzel, U. High-Temperature Tensile Strength of Al10Co25Cr8Fe15Ni36Ti6 Compositionally Complex Alloy (High-Entropy Alloy). *Jom* **67**, 2271-2277 (2015).

8 Kumar, V. A. *et al.* Effect of Test Temperature on Tensile Behavior of Ti-5Al-5V-2Mo-1Cr-1Fe (α plus β) Titanium Alloy with Initial Microstructures in Hot Forged and Heat Treated Conditions. *Metall. Mater. Trans. A* **50A**, 2702-2719 (2019).

9 *Special Metals, Inconel alloy 718*, https://www.specialmetals.com/documents/technical-bulletins/inconel/inconel-alloy-718.pdf (2007).

10 *Haynes International, Haynes Waspaloy alloy*, https://www.haynesintl.com/wp-content/uploads/2023/06/waspaloy.pdf (2017).

11 Kaufman, M. Properties of cast Mar-M-247 for turbine blisk applications. *Superalloys*, 43-52 (1984).

12 Sengupta, A. *et al.* Tensile Behavior of a New Single-Crystal Nickel-Based Superalloy (CMSX-4) at Room and Elevated Temperature. *J. Mater. Eng. Perform.* **3**, 73-81 (1994).

13 Borges, P. P., Ritchie, R. O. & Asta, M. Local lattice distortions and the structural instabilities in bcc Nb-Ta-Ti-Hf high-entropy alloys: An ab initio computational study. *Acta Mater.* **262**, 119415 (2024).

14 Sheikh, S. *et al.* Alloy design for intrinsically ductile refractory high-entropy alloys. *J. Appl. Phys.* **120**, 164902 (2016).

15 Wang, S., Ma, E. & Xu, J. New ternary equi-atomic refractory medium-entropy alloys with tensile ductility: Hafnium versus titanium into NbTa-based solution. *Intermetallics* **107**, 15-23 (2019).

16 Zhang, C. *et al.* Cold-workable refractory complex concentrated alloys with tunable microstructure and good room-temperature tensile behavior. *Scr. Mater.* **188**, 16-20 (2020).

17 Gu, P., Qi, T., Chen, L., Ge, T. & Ren, X. Manufacturing and analysis of

VNbMoTaW refractory high-entropy alloy fabricated by selective laser melting. *Int. J. Refract. Met. Hard Mater.* **105**, 105834 (2022).
18 Li, Q. *et al.* Comparative study of the microstructures and mechanical properties of laser metal deposited and vacuum arc melted refractory NbMoTa medium-entropy alloy. *Int. J. Refract. Met. Hard Mater.* **88**, 105195 (2020).
19 Cai, J. *et al.* Design and coherent strengthening of ultra-high strength refractory high entropy alloys based on laser additive manufacturing. *Mater. Sci. Eng. A* **886**, 145681 (2023).
20 Gou, S. *et al.* Additive manufacturing of ductile refractory high-entropy alloys via phase engineering. *Acta Mater.* **248**, 118781 (2023).
21 Zhang, Y. *et al.* Strong yet ductile refractory high entropy alloy fabricated via additive manufacturing. *Addit. Manuf.* **81**, 104009 (2024).
22 Zhang, Y. *et al.* Origins of strength stabilities at elevated temperatures in additively manufactured refractory high entropy alloy. *Mater. Sci. Eng. A* **915**, 147225 (2024).
23 Su, B. *et al.* Deformation mechanisms of additively manufactured Hf10Nb12Ti40V38 refractory high-entropy alloy: Dislocation channels and kink bands. *Mater. Sci. Eng. A* **915**, 147247 (2024).
24 Mooraj, S. *et al.* Additive manufacturing of defect-free TiZrNbTa refractory high-entropy alloy with enhanced elastic isotropy via in-situ alloying of elemental powders. *Commun. Mater.* **5**, 14 (2024).
25 Dobbelstein, H., Gurevich, E. L., George, E. P., Ostendorf, A. & Laplanche, G. Laser metal deposition of compositionally graded TiZrNbTa refractory high-entropy alloys using elemental powder blends. *Addit. Manuf.* **25**, 252-262 (2019).
26 Liu, C., Wang, Y., Zhang, Y., Zhang, L. & Wang, L. Deformation mechanisms of additively manufactured TiNbTaZrMo refractory high-entropy alloy: The role of cellular structure. *Int. J. Plast.* **173**, 103884 (2024).
27 Kim, Y. S. *et al.* In-situ alloying of nonequiatomic TiNbMoTaW refractory bio-high entropy alloy via laser powder bed fusion: Achieving suppressed microsegregation and texture formation. *Mater. Des.* **252**, 113824 (2025).
28 Duan, R. *et al.* Additive manufacturing of refractory multi-principal element alloy with ultrahigh-temperature strength via simultaneous enhancements in printability and solid solution hardening. *Addit. Manuf.* **91**, 104340 (2024).
29 Liu, C., Zhang, L., Wang, K. & Wang, L. Improving strength and plasticity via pre-assembled dislocation networks in additively manufactured refractory high entropy alloy. *Acta Mater.* **283**, 120526 (2025).
30 Su, B., Zhu, Y., Cheng, F., Zhang, Y. & Li, Z. Achieving exceptional high-temperature strength and oxidation resistance in an additively manufactured refractory high-entropy alloy via strategic elemental substitutions. *J. Alloys Compd.* **1010**, 177079 (2025).
31 Qu, Z. *et al.* High fatigue resistance in a titanium alloy via near-void-free 3D printing. *Nature* **626**, 999–1004 (2024).
32 Gong, H. *et al.* Influence of defects on mechanical properties of Ti–6Al–4 V components produced by selective laser melting and electron beam melting. *Mater. Des.* **86**, 545-554 (2015).

33 Kasperovich, G., Haubrich, J., Gussone, J. & Requena, G. Correlation between porosity and processing parameters in TiAl6V4 produced by selective laser melting. *Mater. Des.* **105**, 160-170 (2016).
34 Jarlov, A. *et al.* Recent progress in high-entropy alloys for laser powder bed fusion: Design, processing, microstructure, and performance. *Mater. Sci. Eng. R* **161**, 100834 (2024).
35 Zhang, Y. *et al.* High specific yield strength and superior ductility of a lightweight refractory high-entropy alloy prepared by laser additive manufacturing. *Addit. Manuf.* **77**, 103813 (2023).
36 Chen, H. *et al.* Feedstock preparation, microstructures and mechanical properties for laser-based additive manufacturing of steel matrix composites. *Int. Mater. Rev.* **68**, 1192-1244 (2023).
37 Gaudez, S. *et al.* High-resolution reciprocal space mapping reveals dislocation structure evolution during 3D printing. *Addit. Manuf.* **71**, 103602 (2023).
38 Tekumalla, S., Seita, M. & Zaefferer, S. Delineating dislocation structures and residual stresses in additively manufactured alloys. *Acta Mater.* **262**, 119413 (2024).
39 Wang, H. *et al.* Effect of cyclic rapid thermal loadings on the microstructural evolution of a CrMnFeCoNi high-entropy alloy manufactured by selective laser melting. *Acta Mater.* **196**, 609-625 (2020).
40 Bertsch, K. M., de Bellefon, G. M., Kuehl, B. & Thoma, D. J. Origin of dislocation structures in an additively manufactured austenitic stainless steel 316L. *Acta Mater.* **199**, 19-33 (2020).
41 Wang, G. *et al.* The origin of high-density dislocations in additively manufactured metals. *Mater. Res. Lett.* **8**, 283-290 (2020).
42 Xiao, B. *et al.* Superior Strength-Ductility Synergy in TiZrNbVAl High Entropy Alloys via Additive Manufacturing. *Acta Mater.* **31**, 121389 (2025).
43 Rao, S. I. *et al.* Atomistic simulations of dislocations in a model BCC multicomponent concentrated solid solution alloy. *Acta Mater.* **125**, 311-320 (2017).
44 Yan, X., Liaw, P. K. & Zhang, Y. Ultrastrong and ductile BCC high-entropy alloys with low-density via dislocation regulation and nanoprecipitates. *J. Mater. Sci. Technol.* **110**, 109-116 (2022).
45 Wang, F. *et al.* Multiplicity of dislocation pathways in a refractory multiprincipal element alloy. *Science* **370**, 95-101 (2020).
46 Lei, Z. *et al.* Enhanced strength and ductility in a high-entropy alloy via ordered oxygen complexes. *Nature* **563**, 546-550 (2018).
47 Wang, S. *et al.* TiZrHfNb refractory high-entropy alloys with twinning-induced plasticity. *J. Mater. Sci. Technol.* **187**, 72-85 (2024).
48 Wang, S. *et al.* Mechanical instability and tensile properties of TiZrHfNbTa high entropy alloy at cryogenic temperatures. *Acta Mater.* **201**, 517-527 (2020).
49 Zhang, X. *et al.* Unique transitions in uniform elongation and deformation mechanisms of a refractory medium-entropy alloy at cryogenic temperatures. *Int. J. Plast.* **186**, 104245 (2025).

50 T Ungár, I Dragomir, Á Révész & Borbély, A. The contrast factors of dislocations in cubic crystals: the dislocation model of strain anisotropy in practice. *J. Appl. Crystallogr.* **32**, 992–1002. (1999).
51 Dirras, G. *et al.* Microstructural investigation of plastically deformed Ti20Zr20Hf20Nb20Ta20 high entropy alloy by X-ray diffraction and transmission electron microscopy. *Mater. Charact.* **108**, 1-7 (2015).
52 Cui, D. *et al.* Unraveling microstructure and mechanical response of an additively manufactured refractory TiVHfNbMo high-entropy alloy. *Addit. Manuf.* **84**, 104126 (2024).
53 An, Y. *et al.* Additive manufacturing of a strong and ductile oxygen-doped NbTiZr medium-entropy alloy. *Mater. Futures* **4**, 015001 (2025).
54 Witzen, W. A. *et al.* Boundary characterization using 3D mapping of geometrically necessary dislocations in AM Ta microstructure. *J. Mater. Sci.* **57**, 9885-9902 (2022).
55 Zhang, Y. *et al.* Strong yet ductile refractory high entropy alloy fabricated via additive manufacturing. *Addit. Manuf.* **81**, 104009 (2024).
