# Supplementary material for: Intrinsic edge dislocations promote high-temperature strength and ductility in additively manufactured refractory high-entropy alloys
Source: Nat Commun. 2026 Apr 24;17:5688. doi: 10.1038/s41467-026-71995-8 (PMC13319749; doi:10.1038/s41467-026-71995-8)
Supplement: Supplementary file 3 — Transparent Peer Review file [file 41467_2026_71995_MOESM3_ESM.pdf]

nature portfolio

Peer Review File

Intrinsic edge dislocations promote high-temperature strength and ductility in additively manufactured refractory high-entropy alloys

Corresponding Author: Professor Robert Ritchie

This manuscript has been previously reviewed at another journal. This document only contains information relating to versions considered at Nature Communications.

This file contains all reviewer reports in order by version, followed by all author rebuttals in order by version.

Version 0:

Reviewer comments:

Reviewer #1

(Remarks to the Author)
The manuscript presents a method for fabricating Ta-containing RHEAs from elemental powders using laser-directed energy deposition. By avoiding post-processing, the method produces a single BCC phase alloy that is superior to its as-cast counterpart in terms of high-temperature strength (~497 MPa) and ductility (~6 %) at 1000 °C. The review is significantly better and technically sound. We recommend that the authors take into account a few recommendations:

• If I understood correctly, the authors are claiming that the dislocation structure formed during AM is helping the high temperature strength, is that correct? Later the authors show that by annealing the material the dislocation structure vanished (as it should) so this increase in properties would extinct itself after a while at high temperatures, correct? The authors should comment on that. I feel this limits a bit the impact of this study.
• I feel a table with samples names and identifications would help the readability.
• On page 7, line 121, the authors highlight the novelty of their fabrication approach for AMed RHEAs, including (i) the “implementation of backing and transition layers… and (ii) optimization of a remelting process…” However, in theory, applying a controlled thermal gradient should lead to a more stable solidification front, reducing defect formation and, consequently, the dislocation density. Furthermore, the remelting strategy described in the Methods section, used to homogenize the powder feedstock, also serves as a cyclic stress-relief thermal treatment, which should further decrease dislocation density. Therefore, from a theoretical standpoint, the proposed processing strategy appears to contradict the reported observation of a high dislocation density in the as-printed alloy. The authors are encouraged to examine and discuss this apparent contradiction in greater depth. This suggestion aligns with Reviewer 2's comment on unrealistic dislocation density when compared with literature data on AM of HEAs (before redetermination done by the authors during the review).
• Page 18, line 333: “Dislocations multiplication and kink band nucleation occur simultaneously, and it is to ensure that the sufficient strain hardening and compatibility stabilize uniform plastic flow and prevent stress localization”. Please clarify the evidence that reveals that dislocation multiplication and kind band nucleation occur concurrently.
• To facilitate reproduction, the characteristics of the raw powder should be readily available, including particle size distribution of each composition, skeletal and apparent densities, and the flowability of the particles.
• In the Methods, section Powder blends, it is stated that the powder has “good flowability”. However, no evidence is provided. The statement is mainly speculative, based on powder sphericity. Other factors also contribute to flowability.
• How were the powders prepared? I am guessing not via gas-atomization. I suggest describing it in methods.
• Paragraphs are extremely long, making it difficult for the reader to follow. I suggest breaking it down into smaller paragraphs.
• Page 6, line 115, at% should be at. %.
• Page 8, line 160, correct typo: “Beyond above performances, it is of important to note what makes”.
• The quality of the figures is not great. For example, it is hard to read the captions inside Fig. 2d due to low resolution. Perhaps consider placing the captions outside the figure.
• I do not understand the fundamental origin of the dislocations during rapid cooling, this high density is not something that

conventionally appears, how have you targeted this issue?
• The dislocation analysis is critical to several of the claims the authors make. I feel the g.b analysis, which is documented on the sup. Materials should be moved to the main article. I do not understand why most of the dislocations are not being characterized, I assume those are from dislocations that would vanish with the other {110} not contained in this zone axis. With that being said: (a) the most important condition, the one deformed at 1000oC has the worst analysis in my opinion since you are showing a single g.b that would lead to invisibility, as the author know and performed in the other samples, the [111] would be preferred and (b) the analysis presented in some cases capture the minority of the dislocations so I feel searching for a better area/orientation would help.
• Has the following effect been reported in other alloys or this is novel from this work? If has been reported this should be stated more clearly since it it ambiguous "As the temperature 221 increases, thermal vacancies overcome the hindrance of screw dislocations created by 222 jogs/dipoles pinning50; edge dislocations may encounter significant energy barriers 223 during sliding, which has also been identified in the as-cast RHEAs28,31,32. In contrast 224 to fully recrystallized alloys where dislocations primarily originate from source 225 activation51, edge dislocations in AMed Nb40 may also derive from intrinsic 226 dislocations generated during the AM process, which can directly participate in slip and 227 multiplication processes. Therefore, the cooperative movement of intrinsic dislocations 228 in AMed RHEAs plays a critical role in affecting their strengthening mechanisms and 229 mechanical properties."

Reviewer #2

(Remarks to the Author)
I co-reviewed this manuscript with one of the reviewers who provided the listed reports. This is part of the Nature Communications initiative to facilitate training in peer review and to provide appropriate recognition for Early Career Researchers who co-review manuscripts.

Version 1:

Reviewer comments:

Reviewer #1

(Remarks to the Author)
I believe the authors have incorporated all the main suggestions we made, I believe the manuscript can be published in this state.

Reviewer #2

(Remarks to the Author)
I co-reviewed this manuscript with one of the reviewers who provided the listed reports. This is part of the Nature Communications initiative to facilitate training in peer review and to provide appropriate recognition for Early Career Researchers who co-review manuscripts.

Open Access This Peer Review File is licensed under a Creative Commons Attribution 4.0 International License, which permits use, sharing, adaptation, distribution and reproduction in any medium or format, as long as you give appropriate credit to the original author(s) and the source, provide a link to the Creative Commons license, and indicate if changes were made.
In cases where reviewers are anonymous, credit should be given to 'Anonymous Referee' and the source.
The images or other third party material in this Peer Review File are included in the article's Creative Commons license, unless indicated otherwise in a credit line to the material. If material is not included in the article's Creative Commons license and your intended use is not permitted by statutory regulation or exceeds the permitted use, you will need to obtain permission directly from the copyright holder.
To view a copy of this license, visit https://creativecommons.org/licenses/by/4.0/

Response to Nature Communications Reviewers' Comments Letter

(NCOMMS-25-68470-T)

__**Response to the co-review comments of Reviewer #1 and #2**__

1. *If I understood correctly, the authors are claiming that the dislocation structure formed during AM is helping the high temperature strength, is that correct? Later the authors show that by annealing the material the dislocation structure vanished (as it should) so this increase in properties would extinct itself after a while at high temperatures, correct? The authors should comment on that. I feel this limits a bit the impact of this study.*

****

Response:

We thank the reviewer for this insightful comment, which allows us to clarify a key aspect of our work and better articulate its fundamental significance. We have conducted additional experiments to support our statement, and a detailed response is as follows:

(1) Thermal stability of edge dislocations

The reviewer correctly notes that annealing leads to the gradual decrease of *total* dislocation density, which is a common phenomenon in metallic materials exposed to high temperatures over extended periods. Our results are fully consistent with this understanding. As documented in Supplementary Table 4, the dislocation density of AMed $Nb_{40}Ta_{25}Ti_{15}Hf_{15}Zr_5$ (AM-Nb40) and AM-Nb40 specimens after 1000°C-1h heat treatment (HT-AM-Nb40) are ~6.26×10^{13} m^{-2} and ~0.914×10^{13} m^{-2}, respectively.

However, a key finding of our work is that this reduction is highly dislocation-type dependent. While screw and mixed dislocations are efficiently annihilated during annealing, edge dislocations persist and remain structurally stable, as directly evidenced by TEM analysis in both the original and revised Fig. 3. This behavior is consistent with prior reports showing that, in chemically complex BCC RHEAs, edge dislocations can experience substantial solute-induced glide barriers and therefore dominate plastic resistance at elevated temperatures[1-6].

Accordingly, our claim is not that *all* AM-generated dislocations remain stable, but rather that thermally stable edge dislocations selectively retained after high-temperature exposure, play a dominant role in sustaining elevated-temperature strength.

(2) High temperature mechanical performance after annealing

To directly address the reviewer's concern that the strengthening effect might "extinct itself" after thermal exposure, we performed additional high-temperature tensile tests on the 1000 °C-annealed AM-Nb40 (HT-AM-Nb40). These results are now included in Supplementary Fig. 9 (Response Fig. 1). Despite the substantial reduction in total dislocation density, HT-AM-Nb40 exhibits a yield strength of ~354 MPa and an ultimate tensile strength of ~506 MPa at 1000 °C, together with a fracture elongation exceeding 11.5%. The results indicate that the strength of AM-Nb40 shows no significant reduction after the dislocation annihilation induced by the 1 h 1000 °C heat treatment, while ductility is further improved relative to the as-built AM state. Importantly, these strength levels remain significantly higher than those of fully recrystallized (FR-Nb40), partially recrystallized (PR-Nb40)[7], and many benchmark high-temperature materials included in Fig. 2c.

By contrast, in AM-Nb40, the intrinsic edge dislocations retained after high-temperature exposure provide substantial local slip resistance and thus sustain strength at 1000 °C. Sustained uniform plastic deformation requires continuous dislocation multiplication to stabilize plastic flow and prevent stress and strain concentrations. The AM-Nb40 maintains this substantial edge dislocation density and demonstrates continuous dislocation multiplication even at elevated temperatures. The mutual interactions between these intrinsic edge dislocations and deformation-induced dislocations from activated sources significantly enhance the work hardening behavior, thereby improving uniform elongation while maintaining high strength.

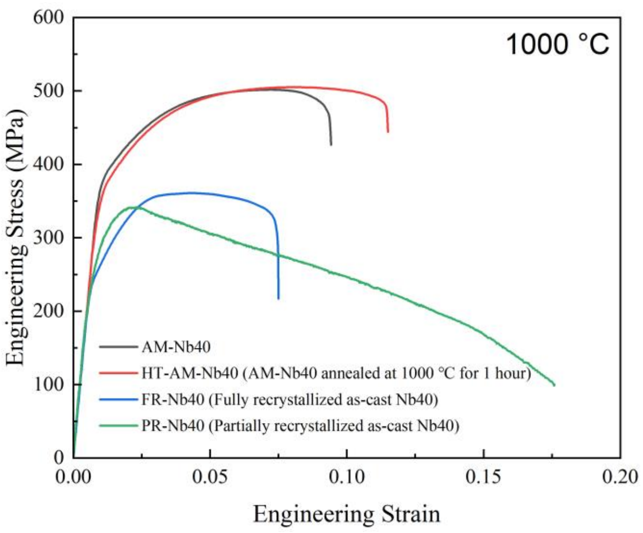

Response Fig. 1 (Supplementary Fig. 9) Tensile engineering stress-strain curves measured at 1000°C of AM-Nb40, HT-AM-Nb40, FR-Nb40, and PR-Nb40.

(3) The novelty and significance of this work

This work is primarily aiming to elucidate the unique strengthening mechanisms enabled by the non-equilibrium microstructure introduced during AM. To the best of our knowledge, the thermal stability of AM-induced edge dislocations and the underlying high-temperature strengthening theory in AMed BCC RHEAs are unprecedented. Previous investigations on the thermal stability of intrinsic dislocation structures induced by additive manufacturing have predominantly limited on AMed FCC alloys containing cellular dislocation networks[8,9]. To the best of our knowledge, a mechanistic analysis of the high-temperature thermal stability and strengthening contribution of intrinsic dislocations in AMed BCC RHEAs has not been reported, and our AM-Nb40 system provides the first such evidence.

Beyond identifying the intrinsic dislocation structure, the broader impact of this work lies in demonstrating that the RHEA with a strong high-temperature strength baseline can be further enhanced through AM processing, and that this advantage can be directly imparted into net-shaped components without post-processing. The resulting strengthening concept is particularly relevant for applications requiring high strength and short-term damage tolerance at elevated temperatures, such as the leading edges of supersonic vehicles.

We agree that long-term service stability is an essential consideration for certain

practical deployment. The present work establishes the mechanistic foundation for this direction, and ongoing efforts are focused on further enhancing the long-term stability of these dislocation structures. Nevertheless, we believe the current findings significantly advance the understanding of how AM can be used not merely as a shaping tool, but as a microstructure-engineering strategy for high-temperature RHEAs.

We hope this clarification addresses the reviewer's concern and demonstrates that the impact of this study extends well beyond transient microstructural effects, providing a new paradigm for designing AM-enabled high-temperature structural materials.

Summary of Revisions Made:

- Included new data for 1000 °C annealed state (HT-AM-Nb40) in Fig. 2c (same as Response Fig. 5c) and Supplementary Table 6 (same as Response Table. 1).
- Revised the main text (the second paragraph on Page 13, the Conclusions paragraph on Page 20).
- Added Supplementary Fig. 9 (Response Fig. 1) in Supplementary Materials.
- Added a description of the new Supplementary Fig. 9 in Supplementary Note 6.

***2.** I feel a table with samples names and identifications would help the readability.*

****

Response:

We thank the reviewer for this excellent and constructive suggestion. We agree entirely that a summary table will significantly enhance the manuscript's clarity and provide readers with a quick reference for the various samples discussed.

In addition to the as-fabricated additively manufactured sample (**AM-Nb40**), we included several reference conditions in this study: fully recrystallized cast Nb40 **(FR-Nb40)**, post-heat-treated AM-Nb40 **(HT-AM-Nb40)**, and the previously reported partially recrystallized cast Nb40 with a heterogeneous lamellar structure **(PR-Nb40)** from Lavernia's group[1]. FR-Nb40 was prepared by arc melting followed by thermomechanical processing to achieve a low initial dislocation density, serving as the primary control for isolating the role of AM-generated dislocations. HT-AM-Nb40 was

annealed and then tensile-tested at 1000 °C to evaluate the thermal stability and mechanical consequences of the AM-introduced defect structure.

Following the reviewer's suggestion, we have (i) **standardized these abbreviations throughout the manuscript**, and (ii) **added a dedicated summary table** compiling the sample nomenclature, processing routes, and corresponding tensile properties (Response Table 1/Supplementary Table 6). For completeness and transparency, we also consolidated the literature comparison data used in Fig. 2c and 2d into the same table. We believe this addition significantly improves the organization and clarity of the revised manuscript.

Response Table 1 (Supplementary Table 6) A summary of tensile properties, including yield strength (σ_y), ultimate tensile strength (σ_t) fracture elongation (ε_f), and uniform elongation (ε_u) of the alloys in Fig. 2c and 2d.

Alloys	Processing Condition	Temperature (°C)	Yield strength (MPa)	Tensile strength (MPa)	Elongation (%)	Uniform elongation (%)	Ref.
AM-Nb40	AM	-50	815.2	898.5	22.6	9.0	This work
			820.8	874.4	16.1	7.0	
			824.4	930.7	16.6	6.5	
		25	743.6	807.3	15.3	5.4	
			767.1	818.1	14.0	4.3	
			730.3	792.6	15.0	5.6	
		900	385.8	528.9	9.7	7.7	
			376.9	501.3	10.5	7.2	
			414.8	538.9	9.5	6.7	
		1000	383.1	501.6	9.4	6.2	
			340.6	484.7	9.7	6.8	
			359.5	505.7	7.5	5.0	
		1100	313.1	414.0	7.3	4.2	
			284.5	396.2	8.2	6.5	
			317.0	417.6	5.6	3.6	
HT-AM-Nb40	AM+1000°C/1h, water quenching	1000	363.3	505.2	11.5	7.2	This work
			344.7	507.6	12.0	8.0	
FR-Nb40	Arc melting + CR85% + 1300°C/1h, water quenching	-50	820.0	852.1	12.5	2.9	This work
		25	781.3	819.0	22.9	9.3	
		900	298.6	416.8	11.0	6.7	
		1000	249.2	360.8	7.5	3.5	
		1100	175.5	236.2	13.5	2.7	
		25	920	1068	14.8	11.6	7

PR-Nb40 (Hetero-structure)	Arc melting + CR90% + 900 °C/1h, water quenching	800	451	550	10.8	2.7	
		900	398	454	11.0	2.6	
		1000	301	325	24.8	1.9	
		1100	180	253	10.8	2.9	
$Nb_{45}Ta_{25}Ti_{15}Hf_{15}$	Arc melting + CR30% + 1100°C/1h, water quenching	25	596.2	641.6	32.1	15.5	10
		800	380.0	443.7	3.2	2.3	
		950	340.8	347.0	2.2	1.1	
		1200	140.2	159.4	15.8	2.0	
CM247LC	As-cast	1000	~500	-	~6.5	<1	11
GH4099	AM	1000	126	-	34.3	<1	12
AlCrCuNiFeCo	As-cast	1000	37	44	77	~2.7	13
AlCoCrFeNiTi	Cast+1220 °C/20h+900°C/5h, air cooling	1000	~130	148	92	~2.0	14
Ti-5Al-5V-2Mo-1Cr-1Fe	Wrought	950	~18	18	146	<2.0	15
NbTaTiHfZr	Wrought	25	964	~1140	16.0	-	16
		800	444	~480	2.0	-	
		1200	30	~125	121.0	-	
Inconel 718	Hot-Rolled Round + Annealed and Aged	25	1124	1365	21	-	17
		538	1020	1193	16	-	
		649	965	1103	15	-	
		760	800	852	5	-	
		871	~309	~309	~64	-	
		982	~140	~140	~171	-	
		1093	~77	~77	~138	-	
HAYNES Waspaloy	Wrought	25	899	1304	24.5	-	18
		538	812	1175	22.0	-	
		649	784	1137	31.9	-	
		760	706	822	32.8	-	
		815.5	517	633	39.7	-	
		871	357	456	48.0	-	
		927	210	297	57.7	-	
		982	132	174	57.8	-	
		1093	31	51	135.5	-	
Mar-M247	Cast + Heat treatment	25	859.8	988.7	5.9	-	19
		538	811.5	1006.6	7.9	-	
		649	783.2	983.2	6.9	-	
		760	808.1	1000.4	4.3	-	
		871	704.6	852.9	3.2	-	
		982	422.0	570.2	6.4	-	

		1038	297.9	407.5	8.3	-	
CMSX-4	Cast + Heat-Treated Condition A	24	888	894	22.0	-	20
		650	907	1031	13.8	-	
		800	916	1151	22.1	-	
		1094	321	459	28.8	-	

Summary of Revisions Made:

- Added all the tensile property data in Supplementary Table 6.
- The abbreviations for different Nb40 reference samples are defined in Methods section.
- Revised the manuscript to ensure the abbreviations for each sample type correspond to those used in Supplementary Table 6.

***3.** On page 7, line 121, the authors highlight the novelty of their fabrication approach for AMed RHEAs, including (i) the "implementation of backing and transition layers… and (ii) optimization of a remelting process…" However, in theory, applying a controlled thermal gradient should lead to a more stable solidification front, reducing defect formation and, consequently, the dislocation density. Furthermore, the remelting strategy described in the Methods section, used to homogenize the powder feedstock, also serves as a cyclic stress-relief thermal treatment, which should further decrease dislocation density. Therefore, from a theoretical standpoint, the proposed processing strategy appears to contradict the reported observation of a high dislocation density in the as-printed alloy. The authors are encouraged to examine and discuss this apparent contradiction in greater depth. This suggestion aligns with Reviewer 2's comment on unrealistic dislocation density when compared with literature data on AM of HEAs (before redetermination done by the authors during the review).*

****

Response:

We sincerely thank the reviewer for this insightful and technically important comment. We fully agree that, **from a classical solidification and annealing perspective**, controlled thermal gradients and remelting might be expected to suppress defect formation and reduce dislocation density. This apparent contradiction is

nontrivial and merits careful discussion. Below, we clarify why **no fundamental contradiction exists** when the full thermomechanical history intrinsic to additive manufacturing (AM) is considered.

Our central clarification is that:

i. The processing strategies employed here do *not* alter the inherently non-equilibrium nature of AM solidification[15,16];

ii. Dislocation evolution in AM is governed by *both* rapid solidification *and* repeated solid-state thermal cycling, rather than by annealing effects alone.

Below, we address these points one by one.

(1) The role of backing and transition layers

The implementation of backing and transition layers served as a critical measure to prevent substrate burn-through and mitigate Ti element segregation. The presence of Ta powder with an ultra-high melting point (3017 °C) in the raw powders necessitates exceptionally high heat input to achieve complete melting. However, excessively high output power can cause burn-through of the substrate and lead to excessive segregation of Ti from the substrate into the RHEA. To prevent burn-through, we deposited six backing layers with intentionally incomplete melting.

Following the backing layers, a transition zone was implemented by gradually increasing heat input to alleviate Ti segregation without stabilizing the solidification front. We acknowledge that our original wording ("thermal gradient management") could be misleading. This terminology may misleadingly associate this process with the solidification characteristics under gentle temperature gradients in classical solidification theory. **In fact, the layer-by-layer nature of LDED results in extreme cooling rates and non-equilibrium solidification process (10^4-10^6 K/s)[21,22]. The progressively increased heat input in the transition zone does not slow down the cooling rate nor establish a stable solidification front.** To prevent confusion, we have revised the manuscript to replace "thermal gradient management" with "**gradually increased heat input**".

(2) Role of remelting: annealing vs. thermal cycling

The remelting strategy offers more opportunities for the melting of high-melting-point refractory alloy elements, which has been repeatedly implemented in research on in-situ multi-metal alloying additive manufacturing[23-26]. We agree with the reviewer that remelting introduces a cyclic thermal exposure that may partially relieve residual stresses and locally annihilate dislocations. However, in AM, **remelting is not a simple annealing process**. Instead, it is superimposed on repeated rapid heating-cooling cycles that induce localized thermal strain and plastic accommodation.

Previous studies have shown that the dislocations typically initially form during rapid solidification and subsequently adapt to thermal deformation through strain localization under cyclic heating[27-30]. This process is analogous to a localized cyclic deformation or tension-compression fatigue, and represents the most significant mechanism for dislocation accumulation in AM alloys[31]. Although each remelting pass may temporarily eliminate some dislocations, the repetitive thermal cycles cause more dynamic accumulation and rearrangement of dislocations, leading to the high initial dislocation density observed in our study.

This phenomenon is well-documented in the AM of refractory alloy. For instance:

- The $Ti_{42}Hf_{21}Nb_{21}V_{16}$ RHEA fabricated via in-situ alloying LDED with layer-by-layer remelting achieved a dislocation density of approximately 6.15×10^{13} m^{-2}, significantly higher than its as-cast counterpart of the same composition[26].
- AMed $Ti_{40}Nb$ alloy subjected to triple remelting per layer exhibited a dislocation density of 9.1×10^{14} m^{-2}, showing no significant difference compared to the single-melted state (9.9×10^{14} m^{-2})[32].
- The MoNbTaW RHEA produced via elementally powder-based LDED retained a high density of cellular dislocation networks even after being subjected to laser remelting with each pass[33].

Furthermore, similar trends are also observed in AMed FCC high-entropy alloys, like CoCrFeNiMn[34] and FeCoCrNi[35], where multi-pass remelting leads to remarked increase of dislocation density. This is attributed to their higher elastic modulus compared to BCC alloys, necessitating greater plastic deformation to accommodate

localized strain after cyclic heating, thereby providing more opportunities for dislocation multiplication[28].

Therefore, the remelting strategy provides increased opportunities for dislocation multiplication within the originally deposited layers through rapid heating and cooling cycles. Moreover, the remelting process parameters in this study were meticulously optimized (Supplementary Note 2) to ensure:

- Complete melting and chemical homogenization.
- Avoiding excessive heat input that would promote large-scale recovery.

This appropriate level of thermal input promotes dislocation multiplication while preventing extensive dislocation annihilation. Consequently, the observed high dislocation density is not contradictory to our processing strategy but rather a direct outcome of it. To better visualize this intrinsic dislocation density, we have added a bright-field (BF) image of the AM-Nb40 sample at low magnification to Fig. 1 of the manuscript (Response Fig. 2).

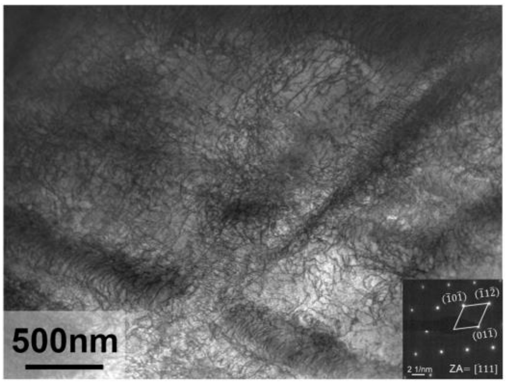

Response Fig. 2 (Revised Fig. 1g) Bright field (BF) TEM image of AM-Nb40 sample reveals a large number of intrinsic dislocations within the material (insert is the selected area electron diffraction).

(3) High dislocation density as an inherent feature in am alloys

To further address concerns regarding the magnitude of the reported dislocation density, we compiled literature data for AM refractory metals and alloys using multiple characterization techniques (XRD-WH, EBSD-KAM, ECCI) (including references cited by Reviewer #2). These data, now summarized in Response Table 2, show that the reported dislocation densities for the AM refractory metals and alloys involved generally exceed 10^{13} m^{-2} and are significantly higher than their as-cast and annealed

counterparts. These dislocation density values are consistent with the reported range and are supported by multiple characterization techniques.

Response Table 2 The dislocation density of some AM metals and alloys.

Refractory alloys	Method	ρ (10^{13} m^{-2})	Ref.
AM-Nb40	XRD	6.26	This work
FR-Nb40	XRD	0.0893	
AM-Nb40 deformed at 1000 °C	XRD	2.53	
HT-AM-Nb40	XRD	0.914	
LDED $Ti_{42}Hf_{21}Nb_{21}V_{16}$	XRD	6.15	26,36
1000 °C anneal state (LDED $Ti_{42}Hf_{21}Nb_{21}V_{16}$)	XRD	1.40	
As-cast $Ti_{42}Hf_{21}Nb_{21}V_{16}$	XRD	1.16	
LPBF TiZrNbTa	XRD	~45	37
As-cast TiZrNbTa	XRD	~18	
LDED $Ti_{41}V_{27}Hf_{13}Nb_{13}Mo_{6}$	EBSD	3	38
LPBF $(NbTiZr)_{99}O_{1}$	EBSD	~5.43	39
LPBF Ti40Nb	XRD	~91	32
LPBF Ti42Nb	ECCI	~1.4	28
LPBF pure Ta	EBSD	≥ 4	40
EBM pure W	EBSD	≥100	41

In summary, the optimized processing strategy and parameters are designed to enhance fabrication quality. The observed high dislocation density is inherently attributed to the thermomechanical history intrinsic to the AM process, and there exists no fundamental contradiction between these observations. Concurrently, we have revised the manuscript to provide a more detailed discussion of these key points and thank the reviewer for raising this important point for clarification.

Summary of Revisions Made:

- A low-magnification bright-field (BF) image has been added as Fig. 1g (Response Fig. 2) to directly illustrate the initial dislocation density in AM-Nb40.
- Revised the figure legends of Fig. 1.
- Added description of the "Laser directed energy deposition processing" in Methods section.
- Added descriptions of the "high intrinsic dislocation density" in Supplementary Note 3 and Supplementary Note 7.
- Added related references to the revised section.

4. Page 18, line 333: "Dislocations multiplication and kink band nucleation occur simultaneously, and it is to ensure that the sufficient strain hardening and compatibility stabilize uniform plastic flow and prevent stress localization". Please clarify the evidence that reveals that dislocation multiplication and kind band nucleation occur concurrently.

****

Response:

We sincerely thank the reviewer for this important comment. We agree that the term "simultaneously" requires clarification, and we appreciate the opportunity to refine both the wording and the supporting evidence. Below, we clarify what is meant by the *concurrent* nature of dislocation multiplication and kink band nucleation, supported by new experimental observations and established physical understanding.

By "concurrency," we do not imply that dislocation multiplication and kink band nucleation are perfectly synchronized in absolute time. Rather, we emphasize that during plastic deformation, they are two continuous, intertwined, and mutually reinforcing processes. Specifically, kink band nucleation does not occur instantaneously; instead, it originates from the progressive accumulation of localized high-density dislocations.

To directly verify this process, we extracted a kink band from the fracture region of a sample tensile-tested at 1000 °C using focused ion beam (FIB) and performed transmission electron microscopy (TEM) analysis. As shown in Response Fig. 3 (Supplementary Fig. 18), the width of this kink band exceeds 2 μm, and its diffraction spots are rotated about the [112] zone axis compared to the matrix (Response Fig. 3c). This demonstrates that the kink band formed by <111>{112} edge dislocations, which is consistent with the intragranular misorientation axis analysis in Response Fig. 4c.

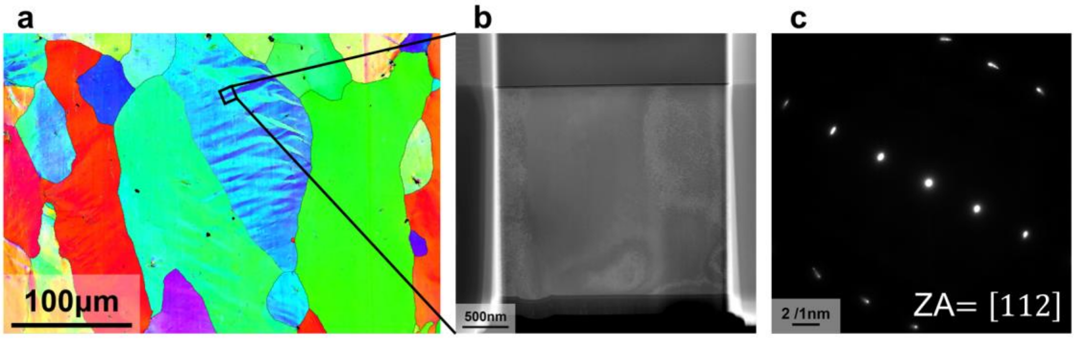

Response Fig. 3 (Supplementary Fig. 18) **Kink bands formed during high-temperature tensile deformation at 1000 °C. a,** EBSD IPF map of tensile sample fractured at 1000 °C, with grain where a FIB lift-out of a kink band was taken. **b,** STEM image of the selected area in **a**. **c,** diffraction pattern of the kink band confirming the [112] zone axis.

Response Fig. 4 is the revised version of Fig. 4a-e in the main text. As shown in Response Fig. 4d, high-density dislocation walls have already been present at the kink band boundaries (indicated by orange arrows), which is consistent with the results reflected by the KAM map (Response Fig. 4b). The dislocation wall are direct products of dislocation slip and multiplication. Simultaneously, the slip band extends to the kink band boundary and terminate there (Response Fig. 4e). Slip bands are macroscopic traces of collective dislocation glide, and their termination at the kink band boundary indicates that dislocation motion is hindered and accumulates at this location.

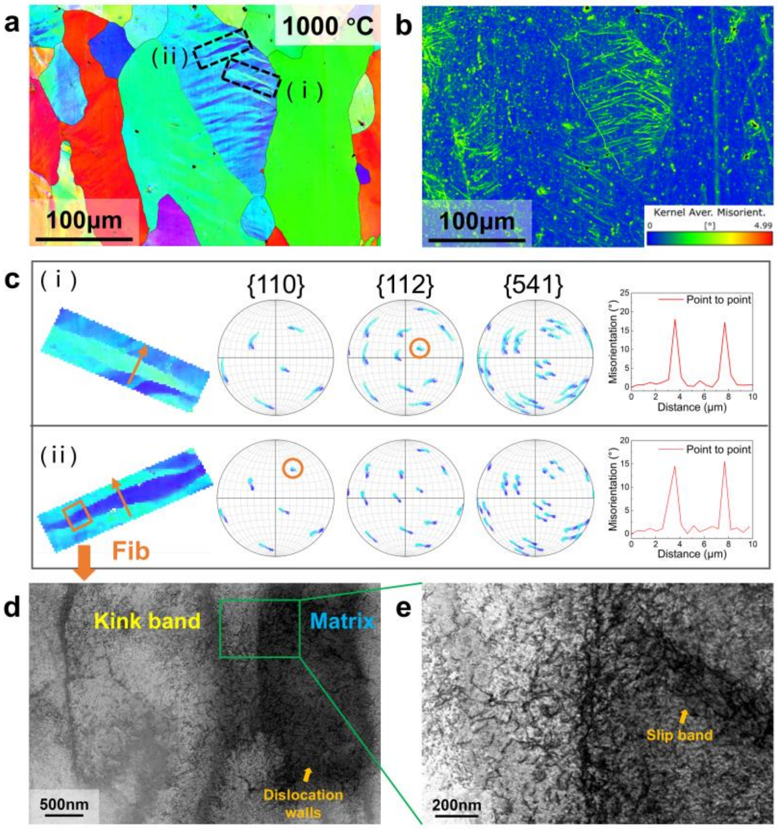

Response Fig. 4 (Revised Fig. 4a-e) **a,** The EBSD IPF+GB map of the tested sample deformed at 1000 °C (Supplementary Fig. 11i) and**. b,** Kernel average misorientation (KAM) map corresponding to **a**. **c,** Two typical kink bands taken from the rectangular subsets in **a**. **d,** The BF TEM image of a segment of the {112} kink band in **c**. **e,** The locally magnified view of the rectangular area in **d**.

Together, these observations demonstrate that **active dislocation multiplication and kink band boundary formation occur over the same deformation stage** even at the high temperature of 1000°C, rather than sequentially in isolated steps. The dislocation walls constitute the embryonic form of the kink band boundary, while continuous dislocation activity (multiplication and accumulation) drives the gradual evolution of this boundary into a mature kink band interface with significant misorientation. This observation aligns with previously reported core views on kink band formation during room-temperature deformation of β-Ti alloys[42,43] and $Ti_{41}V_{27}Hf_{13}Nb_{13}Zr_6$ RHEA[44], collectively supporting the concept of "concurrency."

In summary, thermally stable edge dislocations provide outstanding high-temperature strain hardening, while kink bands also resulting from edge dislocation arrays, continuously rotate the lattice to accommodate the exceptionally high strain near crack tips. These two mechanisms are interdependent during high-temperature

deformation, jointly ensuring sufficient strain hardening and deformation compatibility, thereby stabilizing uniform plastic flow.

Summary of Revisions Made:

- Replace Fig. 4a-e and Supplementary Fig. 11d, 11i, and 11n.
- Add Supplementary Fig. 18 (Response Fig. 3) in the Supplementary.
- Revise the descriptions to avoid ambiguity and modify the corresponding paragraphs in the main text (the first paragraph on Page 16).
- Added references to the revised section for completeness.

5. *Comments on the characteristics of the raw powder (A combined reply to the following three points)*

• *To facilitate reproduction, the characteristics of the raw powder should be readily available, including particle size distribution of each composition, skeletal and apparent densities, and the flowability of the particles.*

• *In the Methods, section Powder blends, it is stated that the powder has "good flowability". However, no evidence is provided. The statement is mainly speculative, based on powder sphericity. Other factors also contribute to flowability.*

• *How were the powders prepared? I am guessing not via gas-atomization. I suggest describing it in methods.*

****

Response:

We are grateful to the reviewer for these constructive and technically important comments regarding the raw powders. We agree that a comprehensive description of the powder characteristics is essential for the reproducibility and scientific rigor of our work. We have thoroughly revised the manuscript to address these points as detailed below.

(1) **Quantitative characterization of powder properties:** We have now conducted a full suite of powder characterization tests to quantitatively describe the powder blends and each elemental powder. The main works are as follows:

• Particle Size Distribution (PSD): The D10, D50, and D90 values were measured according to ASTM B822 standard.

• Densities: The apparent density and tap density were measured according to ASTM B212 and B527 standards.

• Flowability: The flowability was quantitatively assessed using a Hall flowmeter funnel according to ASTM B213, and the results are reported as the flow rate (s/50g).

These data are summarized in Supplementary Table 7 (Response Table 3).

Response Table 3 (**Supplementary Table 7**) Physical property data of raw powder.

Powder feedstock	Particle size (μm)	D10 (μm)	D50 (μm)	D90 (μm)	Tap density (g/cm^3)	Apparent density (g/cm^3)	Flowability (s/50g)
Nb	53-150	68.1	105.2	146.7	5.43	4.21	11.50
Ta	53-150	53.3	89.2	141.2	10.28	9.87	5.49
Ti	53-150	60.1	87.0	126	2.70	2.40	30.31
Hf	53-150	70.7	91.4	121.4	8.39	7.95	7.63
$Zr_{2.5}Nb$	53-150	62.2	92.9	137.3	4.15	3.83	19.7
Powder blends	53-150	61. 7	94.3	135.6	6.42	5.66	12.65

(2) **Revision of flowability description**: Our previous subjective description of powder flowability was inadequate. We have removed the speculative phrase "good flowability". Instead, we now report the quantitative Hall flow rate as mentioned above in Supplementary Table 7 (Response Table 3), providing a more comprehensive and evidence-based assessment of the powder's flow characteristics. The "Methods" section has been modified accordingly.

(3) **Powder production method**: All five types of powders were produced using the Plasma Rotating Electrode Process (PREP). In PREP, the underlying technical principle involves the following steps: within a high-purity inert atmosphere, a plasma arc melts the end of a rapidly rotating electrode bar. The centrifugal force generated by the high-speed rotation then ejects and atomizes the molten metal into fine droplets. Subsequently, these atomized metallic droplets solidify into spherical powder particles within the inert gas environment. This method yields powders with high sphericity, low oxygen content, and narrow size distributions, which are particularly suitable for AM

of refractory alloys. The powder production method has been explicitly stated in the revised "Methods" section.

Summary of Revisions Made:

- Added the particle size distribution, apparent density, tap density, and flowability of the powders before and after mixing in Supplementary Table 7.
- Added the powder production method in Methods section.

6. *Paragraphs are extremely long, making it difficult for the reader to follow. I suggest breaking it down into smaller paragraphs.*

****

Response:

We thank the reviewer for this helpful suggestion. We agree with your point regarding the excessively long paragraphs. In the revised manuscript, we have systematically decomposed the original long paragraphs into shorter paragraphs. Each new paragraph now centers on a single key point, reflecting a clearer logical hierarchy.

Summary of Revisions Made:

- The following paragraph has been split into two paragraphs in main text (the first paragraph on page 7, the second paragraph on page 8, the second paragraph on page 11).
- Newly added paragraphs in main text (the second paragraph on page 13, the first paragraph on page 16).

7. Writing errors (<u>A combined reply to the following two points</u>)

- *Page 6, line 115, at% should be at. %.*
- *Page 8, line 160, correct typo: "Beyond above performances, it is of important to note what makes".*

****

Response:

We have corrected the above errors. The specific corrections are listed below:

Summary of Revisions Made:

• "at%" has been corrected to "at. %" throughout the manuscript

• The sentence has been revised to: "Beyond above performances, what truly distinguishes AM-Nb40 is the observed remarkable strain hardening behavior retained at elevated temperatures."

8. *The quality of the figures is not great. For example, it is hard to read the captions inside Fig. 2d due to low resolution. Perhaps consider placing the captions outside the figure.*

****

Response:

We sincerely thank the reviewer for this valuable suggestion. We fully agree that the clarity of the figures is of utmost importance. **Following the reviewer's recommendation, the captions and labels previously inside the Fig. 2d have been moved to the outside of the figure, and the line width and symbol colors were also adjusted to enhance visual clarity.** The data in Fig. 2c have been also reorganized to incorporate the newly HT-AM-Nb40 (as requested in Comment 1). For transparency and ease of reference, the tensile data from Fig. 2c and 2d have been consolidated into Supplementary Table 6 (Response Table. 1). Furthermore, all figures in the main text have been re-generated with a higher resolution of 600 dpi to ensure all details are sharp and clear. We believe these changes have substantially improved the quality and clarity of our data presentation and we are grateful for the suggestion.

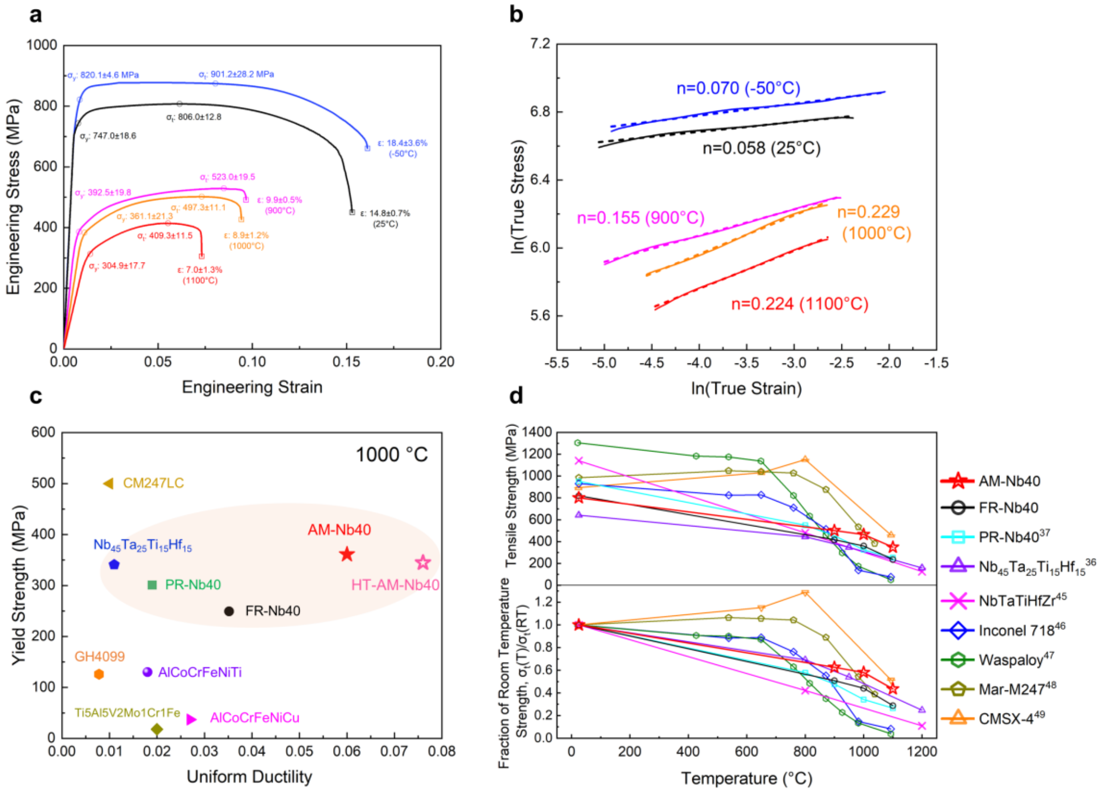

Response Fig. 5 (Revised Fig. 2) **Tensile properties of AM-$Nb_{40}Ta_{25}Ti_{15}Hf_{15}Zr_5$ RHEA. a,** Tensile engineering stress-strain curves measured from -50 °C to 1100 °C. The yield strength (σ_y) and tensile strength (σ_t) are marked on the curves. The tensile specimens refer to Supplementary Fig. 23 for details. **b,** Strain hardening exponents obtained at each temperature. Compared to low temperature properties, the alloy has higher strain hardening exponents at elevated temperatures. **c,** Yield strength *vs*. uniform elongation plots of AM-Nb40, FR-Nb40, other RHEAs with similar compositions, and commercial materials at 1000 °C[7,10-15]. (The testing temperature for $Nb_{45}Ta_{25}Ti_{15}Hf_{15}$ and Ti5Al5V2Mo1Cr1Fe alloys are 950 °C.) The AM-Nb40 has an exceptional synergy of yield strength and uniform elongation at elevated temperatures. **d,** Tensile strength *vs*. temperatures plots of AM-Nb40, commercial Ni-based superalloys and RHEAs[7,10,16-20]. AM-Nb40 has moderate strength and elongation at room temperature, but excellent strength and strength retention at high temperatures. The high-temperature performance is superior to most commercial high-temperature materials. Processing routes and source data for all alloys in **c** and **d** are summarized in Supplementary Table 6.

Summary of Revisions Made:

- The Fig. 2c and 2d (Response Fig. 5c and 5d) have been revised.
- The tensile data from Fig. 2c and 2d have been consolidated into Supplementary Table 6 (Response Table. 1)
- The resolution of all figures in main text has been enhanced.

***9.** I do not understand the fundamental origin of the dislocations during rapid cooling, this high density is not something that conventionally appears, how have you targeted this issue?*

****

Response:

We thank the reviewer for raising this fundamental question. The exceptionally high dislocation density is indeed a distinctive feature of additive manufacturing (AM) processes, which differs significantly from conventional casting routes. Below we clarify (i) the physical origin of these dislocations under AM thermal histories and (ii) how our processing strategy intentionally manages, rather than suppresses this effect.

(1) **Fundamental origin of dislocations in AM:** In AM, dislocations are generated primarily because the material experiences extreme thermal gradients and rapid cooling, followed by repeated localized reheating from subsequent passes/layers. This produces large spatial and temporal gradients in thermal strain. Because the solidified material is constrained by surrounding colder material and the substrate, these thermal strains cannot be accommodated purely elastically and therefore require plastic accommodation, which manifests as dislocation generation and multiplication. More specifically, dislocation accumulation arises from two coupled stages commonly recognized in AM alloys[21-25]:

- Rapid solidification stage: steep thermal gradients and solidification shrinkage introduce high residual stresses and geometrically necessary dislocations to accommodate local incompatibility.
- Solid-state cyclic thermomechanical stage: subsequent layer/pass reheating imposes repeated thermal expansion–contraction cycles, producing localized plastic strain, which further multiplies and rearranges dislocations.

Importantly, this mechanism is fundamentally different from conventional processing routes, where the cooling rates and thermal gradients are far lower and repeated localized reheating is absent; as a result, dislocation densities after casting/annealing are typically orders of magnitude smaller.

(2) **How we "targeted" this issue in the present work:** To date, research on the origin and multiplication of dislocations in AM alloys has predominantly focused on conventional systems exhibiting cellular structures[21,27,29-31,45], with relatively limited studies dedicated to refractory metals and alloys[28]. Our processing strategy was designed to ensure robust fabrication quality (complete melting of Ta-containing feedstock and reduced segregation) while recognizing that **high dislocation density is an inherent consequence of AM thermomechanical history**. Rather than attempting to eliminate dislocations through extensive post-processing, we intentionally retain and harness them as a strengthening reservoir, particularly relevant for BCC RHEAs where edge dislocation character and mobility at elevated temperature are central to strength and strain hardening.

(3) Consistency with literature and validation in our study: The high dislocation density observed in our study has been widely reported in AM BCC alloys. We have compiled dislocation density values from recent literature for various AM refractory metals and alloys, along with some corresponding as-cast and annealed reference data, in Response Table 4. These studies analyzed dislocation density using Electron Channeling Contrast Imaging (ECCI), Kernel Average Misorientation (KAM), and Williamson-Hall (WH) methods. Notably, the reported dislocation densities for the AM refractory metals and alloys involved generally exceed 10^{13} m^{-2} and are significantly higher than their as-cast and annealed counterparts. Our evaluated dislocation density values are consistent with the reported range and are supported by multiple characterization techniques.

Response Table 4 The dislocation density of some AM metals and alloys.

**Refractory alloys**	**Method**	**ρ (10^{13} m^{-2})**	**Ref.**
AM-Nb40	XRD	6.26	This work
FR-Nb40	XRD	0.0893	
AM-Nb40 deformed at 1000 °C	XRD	2.53	
HT-AM-Nb40	XRD	0.914	
LDED $Ti_{42}Hf_{21}Nb_{21}V_{16}$	XRD	6.15	26,36
1000 °C anneal state (LDED $Ti_{42}Hf_{21}Nb_{21}V_{16}$)	XRD	1.40	
As-cast $Ti_{42}Hf_{21}Nb_{21}V_{16}$	XRD	1.16	
LPBF TiZrNbTa	XRD	~45	37
As-cast TiZrNbTa	XRD	~18	
LDED $Ti_{41}V_{27}Hf_{13}Nb_{13}Mo_{6}$	EBSD	3	38
LPBF $(NbTiZr)_{99}O_{1}$	EBSD	~5.43	39
LPBF Ti40Nb	XRD	~91	32
LPBF Ti42Nb	ECCI	~1.4	28
LPBF pure Ta	EBSD	≥ 4	40
EBM pure W	EBSD	≥100	41

We hope this clarification addresses the reviewer's concern and underscores the scientific consensus on the origin of dislocations in AM materials. We are happy to incorporate additional references or discussion on this topic if needed.

Summary of Revisions Made:

- Expanded the discussion on the physical origin of AM-induced dislocations (Supplementary Note 3 and Supplementary Note 7) and added additional references.

10. *The dislocation analysis is critical to several of the claims the authors make. I feel the g.b analysis, which is documented on the sup. Materials should be moved to the main article. I do not understand why most of the dislocations are not being characterized, I assume those are from dislocations that would vanish with the other {110} not contained in this zone axis. With that being said: (a) the most important condition, the one deformed at 1000 ℃ has the worst analysis in my opinion since you are showing a single g.b that would lead to invisibility, as the author know and performed in the other samples, the [111] would be preferred and (b) the analysis presented in some cases capture the minority of the dislocations so I feel searching for a better area/orientation would help.*

****

Response:

We sincerely thank the reviewer for these detailed and technically important comments. We fully agree that the identification of dislocation character is central to several of the key claims in this work, and we appreciate the reviewer's careful scrutiny. In response, we have substantially revised and strengthened the dislocation analysis, as detailed below.

(1) Moving the g·b analysis to the main text

We fully agree that the g·b analysis is central to supporting the identification of dislocation types in this paper. In the revised manuscript, we have fully integrated the BF TEM images under different g vectors from the Supplementary into Fig. 3 (Response Fig. 6). This revision allows readers to directly assess the robustness of the dislocation-type identification without referring to supplementary materials.

(2) Zone-axis selection for the 1000 °C-deformed sample

We fully acknowledge the reviewer's concern regarding the inadequacy of the original analysis for the sample deformed at 1000 °C. In the initial submission, the analysis was performed under a [110] zone axis because the large grain size (>150 μm) of AM-Nb40 limited the availability of thin regions with an optimal orientation. As correctly noted by the reviewer, this resulted in incomplete extinction conditions and weakened the analysis. To address this issue, we have re-prepared TEM specimen and carefully searched for a suitable region. The revised Fig. 3c (Response Fig. 6c) now presents the analysis of 1000 °C-deformed sample consistently under $[1\bar{1}\bar{1}]$ zone axis, which provides the appropriate g·b conditions for distinguishing edge, screw, and mixed dislocations.

(3) Regarding image quality

The clarity of the AM-Nb40 sample in the original Fig. 3a was suboptimal. The specimen has been re-prepared, and a new high-quality BF image has been acquired and is now shown in Fig. 3a (Response Fig. 6a).

(4) Comprehensiveness of dislocation characterization

The reviewer's comment that the "analysis captured only a few dislocations," which has now been corrected. First, by selecting improved zone axes and imaging conditions, the revised analysis captures a substantially larger fraction of dislocations in each condition (Response Fig. 6). Furthermore, we have added an image showing the high overall dislocation density of the AM-Nb40 alloy independent of g·b analysis in Fig. 1 (Response Fig. 2) to represent a global view of its intrinsic dislocation structure.

We believe these revisions have thoroughly addressed the reviewer's concerns, significantly enhancing the reliability and completeness of the dislocation analysis section.

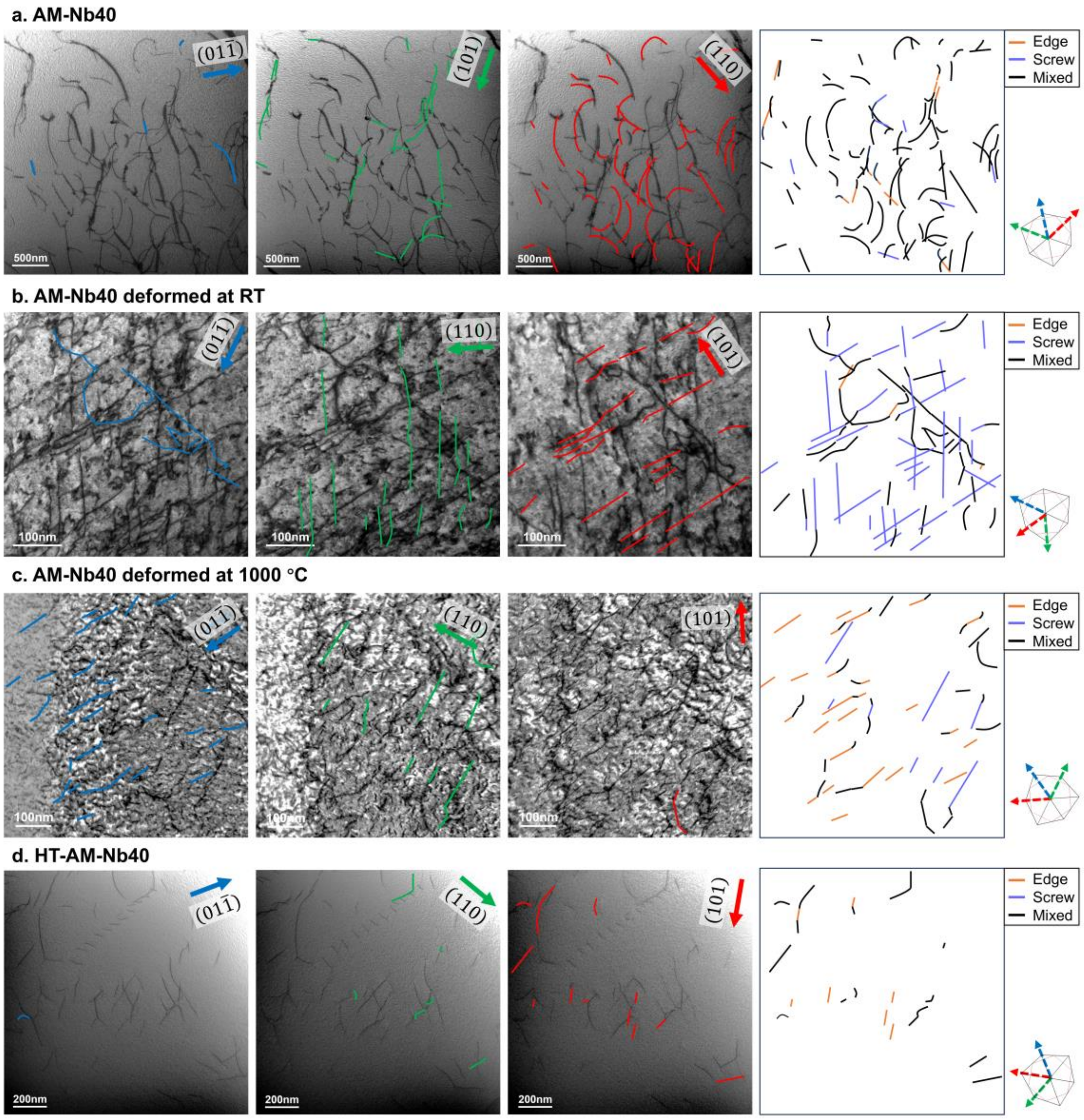

Response Fig. 6 (Revised Fig. 3) **Dislocation types analysis via TEM. a,** Bright field (BF) image of AM-Nb40 sample. **b,c,** BF images of AM-Nb40 RHEA after tensile deformation at room temperature and 1000 °C. **d,** BF image of AM-Nb40 RHEA after annealing at 1000 °C for 1 h (HT-AM-Nb40)**.** The spatial directions of the Burgers vectors are marked on the right side of the images, respectively. Schematics of dislocations colored by type: orange for edge dislocations, purple for screw dislocations, and black for mixed dislocations. Dislocations showing extinction under different g vectors are directly labeled in the corresponding BF micrographs using colors of their Burgers vectors. With increasing deformation temperature, edge dislocations progressively supplant screw dislocations as the dominant feature. The edge dislocations are generated in AM process and maintain stability over a wide temperature range.

Summary of Revisions Made:

- Replaced the analysis data for the AM-Nb40 and 1000 °C deformed samples with newly acquired TEM data in Fig. 3a and 3c (Response Fig. 6a and 6c).

- Moved all g·b extinction-contrast images to the main text from the Supplementary Information.
- Moved the "TEM analysis of dislocation types" to the Method section.
- Added a low-magnification BF-TEM image in Fig. 1 to illustrate the intrinsic dislocation structure.

***11.** Has the following effect been reported in other alloys or this is novel from this work? If has been reported this should be stated more clearly since it is ambiguous "As the temperature increases, thermal vacancies overcome the hindrance of screw dislocations created by jogs/dipoles pinning[50]; edge dislocations may encounter significant energy barriers during sliding, which has also been identified in the as-cast RHEAs[28,31,32]. In contrast to fully recrystallized alloys where dislocations primarily originate from source activation[51], edge dislocations in AMed Nb40 may also derive from intrinsic dislocations generated during the AM process, which can directly participate in slip and multiplication processes. Therefore, the cooperative movement of intrinsic dislocations in AMed RHEAs plays a critical role in affecting their strengthening mechanisms and mechanical properties."*

****

Response:

We sincerely thank the reviewer for this insightful comment. We agree that the original phrasing could be interpreted as ambiguous.

Firstly, the mechanism that edge dislocations can encounter significant energy barriers and play a dominant role in plastic deformation in certain BCC RHEAs, particularly at elevated temperature, has indeed been established in previous works, as we cited (References 29, 32, 33, 52, and 53 in revised main text). This mechanism is therefore not claimed as novel.

The novelty of our work lies instead in demonstrating how this established dislocation physics is uniquely leveraged and amplified in additively manufactured RHEAs, where a high density of AM-generated *intrinsic* dislocations (including edge dislocations) is already present prior to deformation and can directly participate in glide,

interaction, and multiplication. While the thermal stability and strengthening roles of AM-generated dislocation structures have been discussed previously, these investigations have focused primarily on AMed FCC alloys with cellular substructures (References 57-59 in main text). To the best of our knowledge, a mechanistic analysis of the high-temperature thermal stability and strengthening contribution of intrinsic dislocations in AM BCC RHEAs has not been reported, and our AM-Nb40 system provides the first such evidence.

To rectify the ambiguity, we have revised the relevant paragraph in the manuscript accordingly.

Summary of Revisions Made:

- Revised the manuscript text accordingly in Page 11: **"This observation is consistent with previous findings on the temperature-dependent mobility of screw and edge dislocations in as-cast recrystallized alloys[1-3].** As the temperature increases, thermal vacancies overcome the hindrance of screw dislocations created by jogs/dipoles pinning[6]. Meanwhile, edge dislocations may encounter significant energy barriers during sliding. In contrast to fully recrystallized alloys where dislocations primarily originate from source activation[9], edge dislocations in AM-Nb40 may also derive from intrinsic dislocations generated during the AM process, which can directly participate in slip and multiplication processes. Therefore, the cooperative movement of intrinsic dislocations in AM-Nb40 plays a critical role in affecting their strengthening mechanisms and mechanical properties."

References for Response

1 Lee, C. *et al.* Strength can be controlled by edge dislocations in refractory high-entropy alloys. *Nat. Commun.* **12**, 5474 (2021).

2 Wang, F. *et al.* Multiplicity of dislocation pathways in a refractory multiprincipal element alloy. *Science* **370**, 95-101 (2020).

3 Maresca, F. & Curtin, W. A. Mechanistic origin of high strength in refractory BCC high entropy alloys up to 1900K. *Acta Mater.* **182**, 235-249 (2020).

4 Baruffi, C., Maresca, F. & Curtin, W. A. Screw vs. edge dislocation strengthening in body-centered-cubic high entrophy alloys and implications for guided alloy design. *MRS Commun.* **12**, 1111-1118 (2022).

5 Lee, C. *et al.* Temperature dependence of elastic and plastic deformation behavior of a refractory high-entropy alloy. *Sci. Adv.* **6**, eaaz4748 (2020).

6 Rao, S. I., Woodward, C., Akdim, B., Senkov, O. N. & Miracle, D. Theory of solid solution strengthening of BCC Chemically Complex Alloys. *Acta Mater.* **209**, 116758 (2021).

7 Zhang, C. *et al.* Strong and ductile refractory high-entropy alloys with super formability. *Acta Mater.* **245**, 118602 (2023).

8 An, D. *et al.* The Role of Dislocation Type in the Thermal Stability of Cellular Structures in Additively Manufactured Austenitic Stainless Steel. *Adv. Sci.* **11**, 2402962 (2024).

9 Zhang, H., Li, C., Yao, G., Shi, Y. & Zhang, Y. Effect of annealing treatment on microstructure evolution and deformation behavior of 304 L stainless steel made by laser powder bed fusion. *Int. J. Plast.* **155**, 103335 (2022).

10 Cook, D. H. *et al.* Kink bands promote exceptional fracture resistance in a NbTaTiHf refractory medium-entropy alloy. *Science* **384**, 178-184 (2024).

11 Ghoussoub, J. N. *et al.* A new class of alumina-forming superalloy for 3D printing. *Addit. Manuf.* **52**, 102608 (2022).

12 Nie, Z., Guo, Q., Zhao, Y., Ma, Z. & Liu, Y. Temperature dependence tensile behaviors of additively manufactured GH4099 Ni-based superalloy. *Mater. Sci. Eng. A* **899**, 146464 (2024).

13 Kuznetsov, A. V., Shaysultanov, D. G., Stepanov, N. D., Salishchev, G. A. & Senkov, O. N. Tensile properties of an AlCrCuNiFeCo high-entropy alloy in as-cast and wrought conditions. *Mater. Sci. Eng. A* **533**, 107-118 (2012).

14 Daoud, H. M., Manzoni, A. M., Wanderka, N. & Glatzel, U. High-Temperature Tensile Strength of Al10Co25Cr8Fe15Ni36Ti6 Compositionally Complex Alloy (High-Entropy Alloy). *Jom* **67**, 2271-2277 (2015).

15 Kumar, V. A. *et al.* Effect of Test Temperature on Tensile Behavior of Ti-5Al-5V-2Mo-1Cr-1Fe (α plus β) Titanium Alloy with Initial Microstructures in Hot Forged and Heat Treated Conditions. *Metallurgical and Materials Transactions a-Physical Metallurgy and Materials Science* **50A**, 2702-2719 (2019).

16 Mills, L. H. *et al.* Temperature-dependent tensile behavior of the HfNbTaTiZr multi-principal element alloy. *Acta Mater.* **245**, 118618 (2023).

17 *Special Metals, Inconel alloy 718*, https://www.specialmetals.com/documents/technical-bulletins/inconel/inconel-

alloy-718.pdf (2007).
18 *Haynes International, Haynes Waspaloy alloy*, https://www.haynesintl.com/wp-content/uploads/2023/06/waspaloy.pdf (2017).
19 Kaufman, M. Properties of cast Mar-M-247 for turbine blisk applications. *Superalloys*, 43-52 (1984).
20 Sengupta, A. *et al.* Tensile Behavior of a New Single-Crystal Nickel-Based Superalloy (CMSX-4) at Room and Elevated Temperature. *J. Mater. Eng. Perform.* **3**, 73-81 (1994).
21 Chandra, S., Radhakrishnan, J., Huang, S., Wei, S. & Ramamurty, U. Solidification in metal additive manufacturing: challenges, solutions, and opportunities. *Prog. Mater. Sci* **148**, 101361 (2025).
22 Oliveira, J. P., Santos, T. G. & Miranda, R. M. Revisiting fundamental welding concepts to improve additive manufacturing: From theory to practice. *Prog. Mater. Sci* **107**, 100590 (2020).
23 Sing, S. L. *et al.* Emerging metallic systems for additive manufacturing: In-situ alloying and multi-metal processing in laser powder bed fusion. *Prog. Mater. Sci* **119**, 100795 (2021).
24 Pegues, J. W. *et al.* Exploring additive manufacturing as a high-throughput screening tool for multiphase high entropy alloys. *Addit. Manuf.* **37**, 101598 (2021).
25 Dobbelstein, H., Gurevich, E. L., George, E. P., Ostendorf, A. & Laplanche, G. Laser metal deposition of compositionally graded TiZrNbTa refractory high-entropy alloys using elemental powder blends. *Addit. Manuf.* **25**, 252-262 (2019).
26 Zhang, Y. *et al.* Strong yet ductile refractory high entropy alloy fabricated via additive manufacturing. *Addit. Manuf.* **81**, 104009 (2024).
27 Gaudez, S. *et al.* High-resolution reciprocal space mapping reveals dislocation structure evolution during 3D printing. *Addit. Manuf.* **71**, 103602 (2023).
28 Tekumalla, S., Seita, M. & Zaefferer, S. Delineating dislocation structures and residual stresses in additively manufactured alloys. *Acta Mater.* **262**, 119413 (2024).
29 Wang, H. *et al.* Effect of cyclic rapid thermal loadings on the microstructural evolution of a CrMnFeCoNi high-entropy alloy manufactured by selective laser melting. *Acta Mater.* **196**, 609-625 (2020).
30 Bertsch, K. M., de Bellefon, G. M., Kuehl, B. & Thoma, D. J. Origin of dislocation structures in an additively manufactured austenitic stainless steel 316L. *Acta Mater.* **199**, 19-33 (2020).
31 Wang, G. *et al.* The origin of high-density dislocations in additively manufactured metals. *Materials Research Letters* **8**, 283-290 (2020).
32 Subramanian, S. *et al.* Microstructural homogenization through laser remelting in an additively manufactured Ti-40Nb sample from elemental feedstock powders. *J. Mater. Res. Technol.* **38**, 4305-4320 (2025).
33 Moorehead, M. *et al.* High-throughput synthesis of Mo-Nb-Ta-W high-entropy alloys via additive manufacturing. *Mater. Des.* **187**, 108358 (2020).
34 Wang, J. *et al.* Effects of inter-layer remelting frequency on the microstructure

evolution and mechanical properties of equimolar CoCrFeNiMn high entropy alloys during <i>in</i>-<i>situ</i> powder-bed arc additive manufacturing (PBAAM) process. *J. Mater. Sci. Technol.* **113**, 90-104 (2022).

35 Hou, Y. *et al.* Surface re-melting strategy assisted LPBF in-situ alloying technology. *Journal of Manufacturing Processes* **127**, 184-195 (2024).

36 Zhang, Y. *et al.* Origins of strength stabilities at elevated temperatures in additively manufactured refractory high entropy alloy. *Mater. Sci. Eng. A* **915**, 147225 (2024).

37 Mooraj, S. *et al.* Additive manufacturing of defect-free TiZrNbTa refractory high-entropy alloy with enhanced elastic isotropy via in-situ alloying of elemental powders. *Commun. Mater.* **5**, 14 (2024).

38 Cui, D. *et al.* Unraveling microstructure and mechanical response of an additively manufactured refractory TiVHfNbMo high-entropy alloy. *Addit. Manuf.* **84**, 104126 (2024).

39 An, Y. *et al.* Additive manufacturing of a strong and ductile oxygen-doped NbTiZr medium-entropy alloy. *Mater. Futures* **4**, 015001 (2025).

40 Witzen, W. A. *et al.* Boundary characterization using 3D mapping of geometrically necessary dislocations in AM Ta microstructure. *J. Mater. Sci.* **57**, 9885-9902 (2022).

41 Ledford, C. *et al.* Microstructure and high temperature properties of tungsten processed via electron beam melting additive manufacturing*. *Int. J. Refract. Met. Hard Mater.* **113**, 106148 (2023).

42 Li, K., Chen, W., Yu, G., Zhang, J. & Sun, J. A novel deformation mechanism in Ti-V binary metastable β-Ti alloys: Deformation kinking promoted by dislocation accumulation. *J. Alloys Compd.* **875**, 159982 (2021).

43 Li, K. *et al.* Deformation kinking and highly localized nanocrystallization in metastable β-Ti alloys using cold forging. *J. Mater. Sci. Technol.* **120**, 53-64 (2022).

44 Cui, D. *et al.* Kinking induced plasticity in a novel refractory high-entropy alloy. *Vacuum* **227**, 113424 (2024).

45 Gao, L. *et al.* Evolution of dislocations during the rapid solidification in additive manufacturing. *Nat. Commun.* **16**, 4696 (2025).
